# Reassessment of public awareness and prevention strategies for HIV and COVID-19 co-infections through epidemic modeling

**DOI:** 10.1371/journal.pone.0328488

**Published:** 2025-07-31

**Authors:** Dipo Aldila, Joseph Páez Chávez, Bayu Nugroho, Benjamin Idoko Omede, Olumuyiwa James Peter, Putri Zahra Kamalia

**Affiliations:** 1 Department of Mathematics, Faculty of Mathematics and Natural Sciences, Universitas Indonesia, Depok, Indonesia; 2 Innovative Mathematics and Predictive Analytics for Complex System and Technology Laboratory (IMPACT Lab), Universitas Indonesia, Depok, Indonesia; 3 Center for Applied Dynamical Systems and Computational Methods (CADSCOM), Faculty of Natural Sciences and Mathematics, Escuela Superior Politécnica del Litoral, Guayaquil, Ecuador; 4 Center for Dynamics, Department of Mathematics, TU Dresden, Dresden, Germany; 5 Department of Mathematical Sciences, Prince Abubakar Audu (Formerly Kogi State) University, Anyigba, Nigeria; 6 Department of Mathematics, Saveetha School of Engineering, SIMATS, Saveetha University, Chennai, Tamil Nadu, India; 7 Department of Mathematical and Computer Sciences, University of Medical Sciences, Ondo City, Ondo State, Nigeria; 8 Department of Epidemiology and Biostatistics, School of Public Health, University of Medical Sciences, Ondo City, Ondo State, Nigeria; Shanxi University, CHINA

## Abstract

A co–infection model between HIV and COVID-19 that takes into account COVID-19 vaccination and public awareness is discussed in this article. Rigorous analysis of the model is conducted to establish the existence and local stability conditions of the single-infection models. We discover that when the corresponding reproduction number for COVID-19 and HIV exceeds one, the disease continues to exist in both single-infection models. Furthermore, HIV will always be eradicated if its reproduction number is less than one. Nevertheless, this does not apply to the single-infection COVID-19 model. Even when the fundamental reproduction number is less than one, an endemic equilibrium point may exist due to the potential for a backward bifurcation phenomenon. Consequently, in the single-infection COVID-19 model, bistability between the endemic and disease-free equilibrium may arise when the basic reproduction number is less than one. From the co–infection model, we find that the reproduction number of the co–infection model is the maximum value between the reproduction number of HIV and COVID-19. Our numerical continuation experiments on the co–infection model reveal a threshold indicating that both HIV and COVID-19 may coexist within the population. The disease-free equilibrium for both HIV and COVID-19 is stable only if the reproduction numbers are less than one. Additionally, our two-parameter continuation analysis of the bifurcation diagram shows that the condition where both reproduction numbers equal one serves as an organizing center for the dynamic behavior of the co-infection model. An extended version of our model incorporates four different interventions: face mask usage, vaccination, and public awareness for COVID-19, as well as condom use for HIV, formulated as an optimal control problem. The Pontryagin’s Maximum Principle is employed to characterize the optimal control problem, which is solved using a forward-backward iterative method. Numerical investigations of the optimal control model highlight the critical role of a well-designed combination of interventions to achieve optimal reductions in the spread of both HIV and COVID-19.

## 1 Introduction

Human Immunodeficiency Virus (HIV) is a virus that attacks the human immune system, specifically targeting CD4 cells [1]. These cells are essential for the body’s defense ability against infections and illness. Consequently, individuals with HIV are more vulnerable to infections and illnesses compared to healthy individuals. The symptoms experienced by those infected with HIV can range from mild, such as fever, chills, and rash, to severe, including a progressively weakened immune system. If treatment is not received, HIV can progress into Acquired Immunodeficiency Syndrome (AIDS), a more severe and advanced condition where the immune system is severely weakened, making the body susceptible to potentially fatal opportunistic infections and cancers. Early diagnosis and treatment with antiretroviral therapy (ART) are vital in managing the progression of the disease and improving the quality of life for those affected [2]. According to the World Health Organization (WHO), approximately 39.9 million people were living with HIV at the end of 2023, with more than 600,000 deaths recorded in the same year [3]. These figures highlight the ongoing global burden of HIV/AIDS and the critical need for sustained efforts in prevention, early diagnosis, and access to antiretroviral therapy (ART).

In late 2019, the world was struck by a new variant of the coronavirus, known as Severe Acute Respiratory Syndrome Coronavirus 2 (SARS-CoV-2), more commonly referred to as COVID-19. The virus was first detected in Wuhan, China, in late 2019 and rapidly spread worldwide, leading the World Health Organization (WHO) to declare a global pandemic in March 2020 [4]. The virus primarily spreads through respiratory droplets expelled when infected individuals sneeze, cough, talk, or even breathe. It can also spread when susceptible individuals touch contaminated surfaces and then touch their mouth, nose, or eyes [5]. The symptoms of COVID-19 might be moderate or severe. Fever, coughing, exhaustion, loss of taste or smell, and muscle soreness are examples of mild symptoms. Severe symptoms include shortness of breath, confusion, bluish lips or face, and, in some cases, death—particularly among individuals who are immunocompromised or older adults aged 65 years and above. Several methods are available for diagnosing COVID-19, such as Polymerase Chain Reaction (PCR) tests, rapid antigen tests, and imaging techniques like X-rays. Vaccination programs for COVID-19 began worldwide in 2020, offering high efficacy in preventing infection and significantly reducing the severity of symptoms in vaccinated individuals who contract the virus.

As previously explained, HIV can weaken the immune system of infected individuals, making them more susceptible to other diseases. Consequently, co-infection between HIV and COVID-19 is highly likely to occur during the COVID-19 pandemic, as reported in [6, 7]. COVID-19 infected individuals who are already living with HIV appear to have a more severe symptoms to COVID-19, and can even lead to death if not treated properly [8].

Since it was first introduced and formulated in a paper [9], a mathematical model has been used by many authors to model the spread of disease among the population, such as in dengue fever [10–12], malaria [13, 14], tuberculosis [15–19], HIV [20–23], COVID-19 [24–29], and many other types of diseases [30–35]. The concept of the basic reproduction number has been used by these authors as the endemic indicator of their mathematical models. In most cases, the basic reproduction number equal to one becomes the threshold. If the basic reproduction number is larger than one, then there will always exist an endemic equilibrium. On the other hand, there is a chance to eliminate the disease if the basic reproduction number is less than one. The basic reproduction number is defined as the estimated number of secondary cases caused by single primary case in a fully susceptible population. There are several methods that can be used to calculate the basic reproduction number, one of which is the next-generation matrix approach [36].

A mathematical model that describes co-infection between two or more diseases uses a set of mathematical equations to analyze interactions between diseases within the same population. These models often incorporate factors such as disease transmission (both internal and external interactions), recovery, and potential interactions between pathogens. Co-infection models can become complex and require a higher dimension of system due to intricate interactions between diseases. Mathematical models of co-infection involving HIV or COVID-19 with other diseases have been discussed extensively in the literature. Since HIV/AIDS causes immune deficiency, several studies have explored its potential co-infection with other diseases, such as tuberculosis [37–39] and malaria [40, 41]. On the other hand, because COVID-19 emerged only at the end of 2019, co-infection models involving COVID-19 and other diseases have been discussed in more recent studies, such as [42–45] for general co-infections, and specifically for COVID-19 with HIV in [46–50].

The best of our knowledge, the first epidemic model addressing co-infection between HIV and COVID-19 was introduced by the authors in [46], where they developed a six-dimensional system of ordinary differential equations. Their model includes a Susceptible-Infected (SI) compartment for the COVID-19 animal reservoir. Additionally, this article introduces an extended model using fractional-order differential equations. In 2021, the authors in [47] analyzed the model proposed by [46] using an ABC fractional derivative approach. The study explored the phenomenon of backward bifurcation, supported by numerical experiments for the fractional-order model. A more complex co-infection model between HIV and COVID-19 was introduced by the authors in [48]. This model incorporates a vaccinated compartment for COVID-19 and distinguishes between two variants of the virus, the Wild type and the Delta variant, based on their differing infection rates. The article also includes a data fitting process using COVID-19 incidence data and a sensitivity analysis of the model’s reproduction number, employing Partial Rank Correlation Coefficient (PRCC) combined with Latin Hypercube Sampling (LHS). Their findings highlight the significant impact of fractional derivatives on disease dynamics. An optimal control model for the co-infection between HIV and COVID-19 was also discussed by [48]. This eight-dimensional system of ordinary differential equations incorporates vaccination and treatment strategies for COVID-19 and prevention measures for HIV as control variables. The simulations demonstrate that COVID-19 prevention can significantly reduce the burden of co-infections with HIV, and vice versa. Recently, a co-infection model involving HIV, COVID-19, and Monkeypox was explored by the authors in [50]. The study analytically examines single infection models, co-infection between two diseases, and co-infection among three diseases, focusing on the existence of equilibrium points and reproduction numbers. Sensitivity analysis reveals that natural death rates and disease-induced death rates are key parameters influencing the spread of these diseases.

In the post-COVID-19 era, public attention to COVID-19 is no longer as heightened as it was during the pandemic period from 2020 to 2022. COVID-19 booster vaccines are no longer mandatory but have become optional for individuals seeking to enhance their protection against the virus. People who are more aware of COVID-19 are more likely to opt for a vaccine booster. Based on this context, this article presents a co-infection model between HIV and COVID-19 that incorporates population awareness of COVID-19. We conduct a mathematical analysis to examine the existence and stability of equilibrium points, along with the calculation of the reproduction number. Another novel contribution of this study lies in its use of numerical experiments with the continuation software COCO, which is employed to analyze the co-infection model—particularly focusing on the continuation and bifurcation analysis of parameter-dependent equilibria. COCO is chosen for its ability to efficiently track solution branches, detect bifurcation points, and handle large, structured systems, making it well-suited for exploring the rich dynamical behavior inherent in co-infection models. Furthermore, an optimal control extension of the model is analyzed to explore potential strategies for reducing the spread of HIV, COVID-19, and their co-infection. Our optimal control model involved four different interventions, namely the use of face masks, media campaign and vaccination for prevention efforts to reduce the spread COVID-19, and also the use of condom to prevent HIV infection.

The layout of this article is as follows. In Sect 2, we carefully formulate our model based on several key assumptions. The model is constructed as a system of nine-dimensional ordinary differential equations. The model analysis is presented in Sect 3, where we examine the HIV-only model, the COVID-19-only model, and the co-infection model. In Sect 4, we perform a numerical investigation of the co-infection model using COCO. The model extension as an optimal control model is discussed in Sect 5. Finally, Sect 6 provides conclusions and outlines future research directions.

## 2 Construction of the mathematical model

Let the human population be divided into nine compartments based on their health status and type of disease as follows: the susceptible unaware compartment, *S*_*u*_; the susceptible aware compartment, *S*_*A*_; the vaccinated with COVID-19 compartment, *V*; the COVID-19-infected compartment, *C*; the HIV-infected compartment, *H*; the AIDS-infected compartment, *A*; the compartment coinfected with COVID-19 and HIV, *C*_*H*_; the compartment coinfected with COVID-19 and AIDS, *C*_*A*_; and the recovered from COVID-19 compartment, *R*. Since HIV/AIDS is incurable, there is no recovery compartment for individuals with HIV/AIDS. Hence, the total human population, *N*, is given by:


$$N=S_{U}+S_{A}+V+C+H+A+C_{H}+C_{A}+R.$$

**Fig 1:**
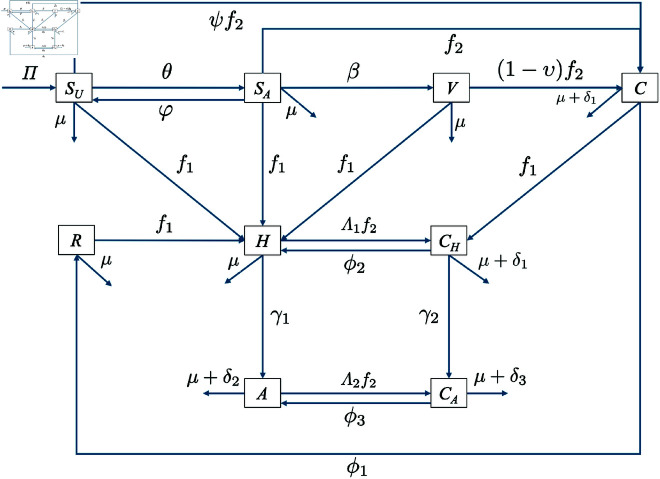
Transmission diagram for the model. The Transmission diagram of Eq (3) is constructed to represent the key epidemiological processes and transitions considered in the study. The system of differential equations is subsequently derived based on the flow structure shown in this diagram.

The model construction follows the transmission diagram shown in Fig 1, with details described as follows. We assume that HIV/AIDS and COVID-19 are not transmitted vertically to newborns; transmission occurs only through direct or close contact between susceptible individuals and infected individuals. Hence, all newborns are assumed to be susceptible and enter the population through the *S*_*U*_ compartment at a constant rate, Π. We incorporate population awareness of COVID-19 into our model. Awareness of HIV/AIDS is not included, as we assume HIV/AIDS is a long-established disease to which the population is already aware. On the other hand, awareness of COVID-19 still needs to be enhanced through media campaigns or other approaches. Susceptible unaware individuals are those who do not fully recognize the dangers of COVID-19 transmission. Consequently, they neither receive the COVID-19 vaccine nor take protective measures against COVID-19 transmission. There is a transition from unaware to aware susceptible individuals at a rate of φ. This awareness is assumed to be temporary; thus, there is a dropout rate from *S*_*A*_ to *S*_*U*_ at a constant rate, θ. Furthermore, only aware susceptible individuals receive the COVID-19 vaccine at a constant rate, β.

HIV/AIDS infection occurs through direct contact between susceptible individuals ($S_{U},S_{A},V,C$, and *R*) and those infected with HIV/AIDS (*H*,*A*,*C*_*H*_ and *C*_*A*_). We assume that the probability of a successful infection with HIV/AIDS is $\chi_{1}$. Therefore, the transmission term for HIV/AIDS is given by

$$f_{1}=\frac{\chi_{1}(H+\omega (A+C_{H}+C_{A}))}{N},$$ (1)

where ω is a correction parameter greater than one, reflecting that individuals in compartments *A*, *C*_*H*_, and *C*_*A*_ are more infectious than those in *H* due to an increased viral load of HIV.

We assume that COVID-19 infection occurs through contact with individuals infected with COVID-19 (*C*) or those coinfected with both COVID-19 and HIV/AIDS, represented by *C*_*H*_ or *C*_*A*_. The standard transmission rate is denoted by $\chi_{2}$. Thus, the infection term for COVID-19 is given by

$$f_{2}=\frac{\chi_{2}(C+\eta (C_{H}+C_{A}))}{N},$$ (2)

where η<1 is a correction parameter for COVID-19 infection due to contact with coinfected individuals. We assume that η is less than one because individuals infected with HIV/AIDS are unable to engage in normal social activities like those who are not infected with HIV/AIDS. An important feature of our model is that individuals infected with HIV or AIDS have a higher likelihood of contracting COVID-19 due to their weakened immune systems. Therefore, we introduce correction parameters $\Lambda_{1}$ and $\Lambda_{2}$, where $\Lambda_{2}>\Lambda_{1}>1$, corresponding to increased susceptibility to COVID-19 for individuals in compartments *H* and *A*, respectively. Hence the co–infection term of *H* and *A* compartment is given by $\Lambda_{1}f_{2}$ and $\Lambda_{2}f_{2}$, respectively.

Due to varying levels of awareness and vaccination within the susceptible population, the probability of successful COVID-19 transmission differs between compartments *S*_*U*_, *S*_*A*_, and *V*. The transmission rate for the vaccinated compartment, *V*, is the lowest, while *S*_*U*_ has the highest transmission rate. Thus, new infections from the *S*_*U*_ compartment are given by $\psi f_{2}S_{U}$, where ψ>1 is the correction parameter for the infection term for *S*_*U*_ individuals. For the *S*_*A*_ compartment, it is given by $f_{2}S_{A}$, and for the *V* compartment, it is $(1\;-\;\upsilon )f_{2}V$, where υ represents COVID-19 vaccine efficacy.

An additional key assumption in our model is that COVID-19 and AIDS may cause death at constant rates, denoted by $\delta_{1}$, $\delta_{2}$, and $\delta_{3}$ for individuals in compartments *C*/*C*_*H*_, *A*, and *C*_*A*_, respectively. Since COVID-19 is a recurrent disease, we define $\phi_{1}$, $\phi_{2}$, and $\phi_{3}$ as the recovery rates for individuals in compartments *C*, *C*_*H*_, and *C*_*A*_, respectively. Moreover, the transition rate from *H* to *A*, representing the progression of HIV infection, is denoted by $\gamma_{1}$, while the transition rate from *C*_*H*_ to *C*_*A*_ is represented by $\gamma_{2}$, with $\gamma_{2}>\gamma_{1}$.

Based on above description, the mathematical model for co–infection between HIV/AIDS and COVID-19 is given as follows:

$$S_{U}^{\prime }=\Pi -(f_{1}+\psi f_{2}+\mu +\theta )S_{U}+\varphi S_{A},\;S_{A}^{\prime }=\theta S_{U}-(f_{1}+f_{2}+\mu +\varphi +\beta )S_{A},\;V^{\prime }=\beta S_{A}-(f_{1}+(1-\nu )f_{2}+\mu )V,\;C^{\prime }=\psi f_{2}S_{U}+f_{2}S_{A}+(1-\nu )f_{2}V-(f_{1}+\phi_{1}+\mu +\delta_{1})C,\;R^{\prime }=\phi_{1}C-(f_{1}+\mu )R,\;H^{\prime }=f_{1}S_{U}+f_{1}S_{A}+f_{1}V+f_{1}R+\phi_{2}C_{H}-(\mu +\Lambda_{1}f_{2}+\gamma_{1})H,\;A^{\prime }=\gamma_{1}H+\phi_{3}C_{A}-(\Lambda_{2}f_{2}+\mu +\delta_{2})A,\;C_{H}^{\prime }=f_{1}C+\Lambda_{1}f_{2}H-(\phi_{2}+\gamma_{2}+\mu +\delta_{1})C_{H},\;C_{A}^{\prime }=\gamma_{2}C_{H}+\Lambda_{2}f_{2}A-(\phi_{3}+\mu +\delta_{3})C_{A},$$ (3)

where *f*_1_ and *f*_2_ is the infection term for HIV/AIDS and COVID-19 given in Eqs (1) and (2), respectively. The description of model parameter is given in Table 1.

**Table 1:** Parameter definitions for model (3).

Par.	Definition	Values	Ref
Π	Recruitment rate of the human population	$\frac{10000}{73\times 365}$	Assumed
μ	Natural death rate of the human population	$\frac{1}{73\times 365}$	Assumed
	**HIV/AIDS model parameters**		
$\chi_{1}$	HIV infection rate	0.18	[23]
ω	Correction parameter for $\chi_{1}$, representing that individuals in *A*, *C*_*H*_, and *C*_*A*_ have greater access to susceptible individuals due to milder HIV symptoms compared to individuals in *H*	ω>1	Assumed
$\gamma_{1}$	Progression rate from HIV to AIDS due to increased viral load	0.07	[23]
$\delta_{2}$	Death rate due to AIDS	0.016	[23]
	**COVID-19 model parameters**		
$\chi_{2}$	COVID-19 infection rate	0.88	[51]
η	Reduction in $\chi_{2}$ due to co-infection of HIV/AIDS and COVID-19 in individuals *C*_*H*_ and *C*_*A*_	(0,1)	Assumed
ψ	Correction parameter for $\chi_{2}$ due to unawareness in individuals in *S*_*U*_	ψ>1	Assumed
θ	Media campaign rate to increase public awareness of COVID-19	(0,1)	Assumed
φ	Dropout rate due to loss of COVID-19 awareness	(0,1)	Assumed
β	COVID-19 vaccination rate	(0,1)	Assumed
ν	COVID-19 vaccine efficacy	0.8	[52]
$\phi_{1}$	Recovery rate from COVID-19 for individuals in *C*	0.1	[24]
	**Co-infection model parameters**		
$\Lambda_{1}$	Correction parameter representing increased transmissibility of COVID-19 in individuals with HIV (*H*)	$\Lambda_{1}>1$	Assumed
$\Lambda_{2}$	Correction parameter representing increased transmissibility of COVID-19 in individuals with AIDS (*A*)	$\Lambda_{2}>1$	Assumed
$\phi_{2}$	Recovery rate from COVID-19 for individuals in *C*_*H*_	$\frac{1}{15}$	[24]
$\phi_{3}$	Recovery rate from COVID-19 for individuals in *C*_*A*_	$\frac{1}{15}$	[24]
$\gamma_{2}$	Progression rate from *C*_*H*_ to *C*_*A*_ due to increased viral load	$\frac{0.2}{365}$	Assumed
$\delta_{1}$	Death rate due to co-infection of COVID-19 and HIV	0.018	[52]
$\delta_{3}$	Death rate due to co-infection of COVID-19 and AIDS	0.01843	Assumed

## 3 Model analysis

In this section, we discuss the basic reproduction number of our model, which is a critical threshold parameter determining whether an infectious disease can invade and persist in a population. Given the complexity of the co–infection model, which simultaneously incorporates both HIV/AIDS and COVID-19 dynamics, an analytical examination of becomes highly intricate. To facilitate a more manageable analysis, we begin by exploring the non-co–infection models—specifically, the HIV/AIDS-only model (Sect 3.1 and the COVID-19-only model (Sect 3.2). By deriving and analyzing for these simplified scenarios, we gain valuable insights into the individual disease dynamics. These insights serve as a foundation for anticipating the potential behavioral patterns and outcomes of the more complex co–infection model, thereby enhancing our understanding of its possible dynamic outputs.

### 3.1 HIV only model

**Fig 2:**
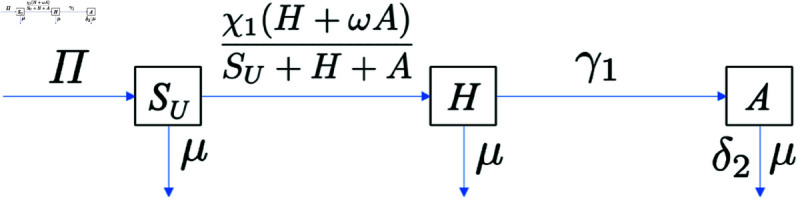
HIV/AIDS-only transmission diagram. Transmission diagram of HIV/AIDS-only model in system (4).

Without COVID-19 co-infection, the total population is given by $N_{h}=S_{U}+H+A$. The transmission diagram of the mathematical model for HIV-only infection, based on model (3), is shown in Fig 2, and the model is presented as follows:

$$S_{U}^{\prime }=\Pi -\frac{\chi_{1}(H+\omega A)}{S_{U}+H+A}S_{U}-\mu S_{U},\;H^{\prime }=\frac{\chi_{1}(H+\omega A)}{S_{U}+H+A}S_{U}-\gamma_{1}H-\mu H,\;A^{\prime }=\gamma_{1}H-\mu A-\delta_{2}A.$$ (4)

Since the above model represents the human population, it is necessary that all variables in model (4) are non-negative for all *t* > 0. This properties is discussed in the following lemma.

**Lemma 1.** *If the initial conditions of system (4) given by $S_{U}(0)>0,H(0)\geq 0,A\geq 0$, then the solutions *S**_*U*_*(*t*),*H*(*t*), and A(t) of system (4) are non-negative for all time t > 0.*

*Proof:* In the boundary of ${\mathbb{R}}_{+}^{3}$, we have:


$$S_{U}^{\prime }(S_{U}=0)=\Pi >0,\;\;\;\;\;H^{\prime }(H=0)=\frac{\chi_{1}(H+\omega A)}{S_{U}+A}S_{U}\geq 0,\;\;\;\;\;A^{\prime }(A=0)=\gamma_{1}H\geq 0.$$

From the above calculation, it can be seen that all directions on the boundary are always pushed back inward to ${\mathbb{R}}_{+}^{3}$. Hence, since the initial conditions are always non-negative in ${\mathbb{R}}_{+}^{3}$, the solutions *S*_*U*_(*t*), *H*(*t*), and *A*(*t*) remain positive for all time *t* > 0. Therefore, the proof is complete. ◻

**Lemma 2.** *The feasible region defined by*


$$\Omega_{HIV}=\left\{(S_{U},H,A)\in {\mathbb{R}}_{+}^{3}:S_{U}+H+A=N_{h}\leq \max\left\{\frac{\Pi }{\mu },N_{h}(0)\right\}\right\},$$

*is positively invariant and attracting with regard of system (4).*

*Proof:* Since the initial conditions is non-negative, then it is sufficient to prove that *S*_*U*_(*t*),*H*(*t*),*A*(*t*) is bounded in ${\mathbb{R}}_{+}^{3}$. By summing all equation in (4) we have:


$$N_{h}^{\prime }=S_{U}^{\prime }+H^{\prime }+A^{\prime }=\Pi -\mu N_{h}-\delta_{2}A<\Pi -\mu N_{h}.$$

Hence, the solution will satisfy $N_{h}(t)\leq \frac{\Pi }{\mu }+\left(N_{h}(0)-\frac{\Pi }{\mu }\right)e^{-ct}$, implying $\lim_{t\to \infty }N_{h}(t)=\frac{\Pi }{\mu }$. Furthermore, it is always satisfy $N_{h}(t)\in \max\left\{\frac{\Pi }{\mu },N_{h}(0)\right\}$. Therefore, the differential equation $\frac{dN_{h}}{dt}\leq \Pi -\mu N_{h}$ has the solution that is always bounded in the domain Ω. Hence the proof is complete. ◻

#### 3.1.1 HIV-free equilibrium point and the basic reproduction number ${ℛ}_{0_{HIV}}$.

Taking the right hand side of system (4) equal to zero, and solve it respect to susceptible *S*_*U*_by assuming *H* = *A* = 0, the disease-free equilibrium for HIV only model in Eq 4 is given by:

$${ℰ}_{HIV}^{0}=\left(S_{U},H,A\right)=\left(\frac{\Pi }{\mu },0,0\right).$$ (5)

In the context of an HIV infection model, the disease-free equilibrium (${ℰ}_{HIV}^{0}$) represents a critical theoretical state where no individuals are infected with HIV in the population — that is, all infection-related compartments (e.g., HIV and AIDS compartment) are at zero, and only susceptible compartment have nonzero populations. Furthermore, ${ℰ}_{HIV}^{0}$ provides the baseline for assessing control strategies through the calculation of the respected basic reproduction number.

Next we calculate the basic reproduction number of the HIV-only model in (4). The basic reproduction number, representing the potential number of secondary cases caused by a single primary case in a completely susceptible population during the infectious period of the primary case, has been widely used by many researchers to determine the qualitative behavior of their epidemiological models, including whether the disease will disappear or persist. In our case, we calculate the basic reproduction number (denoted by ${ℛ}_{0_{HIV}}$) using the next-generation matrix approach [36]. The transition (*F*_*h*_) and transmission $\left(V_{h}\right)$ matrix from infected compartment *H* and *A* of model (4) is given by:


$$F_{h}=\left[\begin{array}{cc} -\gamma_{1}-\mu & 0\\ \gamma_{1} & -\mu -\delta_{2} \end{array}\right],\;\;\;\;\;\;\;\;V_{h}=\left[\begin{array}{cc} \chi_{1} & \chi_{1}\omega \\ 0 & 0 \end{array}\right].$$

Since there is a zero row of $V_{h}$, then the next-generation matrix formula is given by $K_{h}=-E^{\prime }FV^{-1}E,$ where E=[10]. Hence, the next-generation matrix is given by:


$$K_{h}=\left[\frac{\chi_{1}(\omega \gamma_{1}+\mu +\delta_{2})}{\left(\gamma_{1}+\mu )(\delta_{2}+\mu \right)}\right].$$

Therefore, the basic reproduction of the HIV-only model is taken from the spectral radius of *K*_*h*_, and given by:

$${ℛ}_{0_{HIV}}=\frac{\chi_{1}(\omega \gamma_{1}+\mu +\delta_{2})}{\left(\gamma_{1}+\mu )(\delta_{2}+\mu \right)}.$$ (6)

#### 3.1.2 The HIV-endemic equilibrium point.

The second equilibrium of the HIV-only model in (4) is the endemic equilibrium point, where all compartment in model (4) exist in the equilibrium state. This equilibrium is denoted by ${ℰ}_{HIV}^{1}$ and is given by:

$${ℰ}_{HIV}^{1}=(S_{U},H,A)=\left(S_{U}^{†},H^{†},A^{†}\right),$$ (7)

where:


$$S_{U}^{†}=\frac{\Pi (\mu +\delta_{2}+\gamma_{1})}{\delta_{2}\gamma_{1}({ℛ}_{0_{HIV}}-1)+\mu {ℛ}_{0_{HIV}}(\mu +\delta_{2}+\gamma_{1})},$$

$$H^{†}=\frac{\Pi (\mu +\delta_{2})({ℛ}_{0_{HIV}}-1)}{\delta_{2}\gamma_{1}({ℛ}_{0_{HIV}}-1)+\mu {ℛ}_{0_{HIV}}(\mu +\delta_{2}+\gamma_{1})},$$

$$A^{†}=\frac{\Pi \gamma_{1}({ℛ}_{0_{HIV}}-1)}{\delta_{2}\gamma_{1}({ℛ}_{0_{HIV}}-1)+\mu {ℛ}_{0_{HIV}}(\mu +\delta_{2}+\gamma_{1})}.$$

Based on the above expression, we have the following theorem regarding the existence of HIV-endemic equilibrium point:

**Theorem 1.** *The HIV-endemic equilibrium point of model (4), denoted by ${ℰ}_{HIV}^{1}$ exist only if ${ℛ}_{0_{HIV}}>1$ and not exist otherwise, where ${ℛ}_{0_{HIV}}$ is the basic reproduction number of the HIV-only model in (4).*

#### 3.1.3 The stability of the HIV-free equilibrium point.

The linearization of model (4) in ${ℰ}_{HIV}^{0}$ is given by:


$$J({ℰ}_{HIV}^{0})=\left[\begin{array}{ccc} -\mu & -\chi_{1} & -\chi_{1}\omega \\ 0 & \chi_{1}-\gamma_{1}-\mu & \chi_{1}\omega \\ 0 & \gamma_{1} & -\mu -\delta_{2} \end{array}\right].$$

We can see clearly that the first column of $J({ℰ}_{HIV}^{0})$ is −μ. Hence, λ=−μ is the first eigenvalues of $J({ℰ}_{HIV}^{0})$. The other eigenvalues is taken from the block matrix 2×2 matrix that was left. The characteristic polynomial for the other two eigenvalues is given by:


$$f(\lambda ,{ℛ}_{0_{HIV}})=\lambda^{2}+(2\mu +\gamma_{1}+\delta_{2}-\chi_{1})\lambda +(\mu +\gamma_{1})(\mu +\delta_{2})(1-{ℛ}_{0_{HIV}})=0.$$

Since


$$\frac{\chi_{1}}{2\mu +\delta_{2}+\gamma_{1}}<\frac{\chi_{1}}{\mu +\gamma_{1}}<\frac{\chi_{1}(\omega \gamma_{1}+\mu +\delta_{2})}{\left(\gamma_{1}+\mu )(\delta_{2}+\mu \right)}={ℛ}_{0_{HIV}},$$

then all coefficient of $f(\lambda ,{ℛ}_{0_{HIV}})$ will be positive if ${ℛ}_{0_{HIV}}<1$. Hence, we have the following theorem regarding the local stability criteria of ${ℰ}_{HIV}^{0}$.

**Theorem 2.** *The HIV-free equilibrium ${ℰ}_{HIV}^{0}$ is locally asymptotically stable if ${ℛ}_{0_{HIV}}<1$, and unstable if ${ℛ}_{0_{HIV}}>1$.*

#### 3.1.4 The stability of HIV-endemic equilibrium point.

We use Castillo-Song bifurcation theorem [53] to analyze the stability of equilibria around ${ℛ}_{0_{HIV}}=1$. First, we rewrite our model in (4) as follows:

$$h_{1}=\Pi -\frac{\chi_{1}(x_{2}+\omega x_{3})}{x_{1}+x_{2}+x_{3}}x_{1}-\mu x_{1},$$ (8a)

$$h_{2}=\frac{\chi_{1}(x_{2}+\omega x_{3})}{x_{1}+x_{2}+x_{3}}x_{1}-(\gamma_{1}+\mu )x_{2},$$ (8b)

$$h_{3}=\gamma_{1}x_{2}-(\mu +\delta_{2})x_{3}.$$ (8c)

Let the bifurcation parameter is $\chi_{1}$ evaluated at ${ℛ}_{0_{HIV}}=1$, gives:


$$\chi_{1}^{*}=\frac{\left(\mu +\gamma_{1})(\mu +\delta_{2}\right)}{\mu +\delta_{2}+\omega \gamma_{1}}.$$

Evaluate the Jacobian matrix of 8*a* in ${ℰ}_{HIV}^{0}$ and $\chi_{1}^{*}$ has three eigenvalues, i.e. $\lambda_{1}=0$, $\lambda_{2}=-\mu$, and $\lambda_{3}=-\frac{\omega \gamma_{1}(2\mu +\delta_{2}+\omega_{1})+(\mu +\delta_{2}){\;}^{2}}{\omega \gamma_{1}+\mu +\delta_{2}}$. Since we have a simple zero eigenvalue, while the other two eigenvalues are negative, then we can use the Castillo-Song bifurcation theorem.

In Castillo-Song bifurcation theorem, we need to calculate two bifurcation type indicators, namely 𝒜 and ℬ using the following recepies:

$$𝒜=\sum_{k,i,j=1}^{3}\left(v_{k}w_{i}w_{j}\frac{\partial^{2}h_{k}}{\partial x_{i}\partial x_{j}}({ℰ}_{HIV}^{0})\right),\;\;\;\;\;ℬ=\sum_{k,i=1}^{3}\left(v_{k}w_{j}\frac{\partial^{2}h_{k}}{\partial x_{i}\partial \chi_{1}}({ℰ}_{HIV}^{0})\right),$$ (9)

where $w=[w_{1}\;\;w_{2}\;\;w_{3}{]}^{T}$ and $v=[v_{1}\;\;v_{2}\;\;v_{3}]$ is the right and left eigenvector respected to λ=0. By direct calculation, we have:


$$w_{1}=\frac{w_{3}(\mu +\gamma_{1})(\mu +\delta_{2})}{\gamma_{1}\mu },\;\;\;\;\;\;\;w_{2}=\frac{w_{3}(\mu +\delta_{2})}{\gamma_{1}},\;\;\;\;\;\;\;w_{3}=w_{3}>0,$$

$$v_{1}=0,\;\;\;\;\;\;\;v_{2}=v_{2}>0,\;\;\;\;\;\;\;v_{3}=\frac{v_{2}\omega (\mu +\gamma_{1})}{\omega \gamma_{1}+\mu +\delta_{2}}.$$

Substituting $v_{i}$ and *w*_*i*_ to 𝒜 and ℬ, yields:


$$𝒜=-\frac{2\omega w_{3}^{2}\chi_{1}^{*}\mu (\mu +\gamma_{1}+\delta_{2})(\omega \gamma_{1}+\mu +\delta_{2})}{\Pi \gamma_{1}^{2}}<0,$$

$$ℬ=\frac{v_{2}w_{3}(\omega \gamma_{1}+\mu +\delta_{2})}{\gamma_{1}}>0.$$

Since 𝒜<0 and ℬ>0, then we have that system 8*a* always exhibit a forward bifurcation at ${ℛ}_{0_{HIV}}=1$. Hence, we have the following theorem.

**Theorem 3.** *The HIV-endemic equilibrium point ${ℰ}_{HIV}^{1}$ of system (4) is locally asymptotically stable for ${ℛ}_{0_{HIV}}>1$, but close to one.*

### 3.2 COVID-19 only model

**Fig 3:**
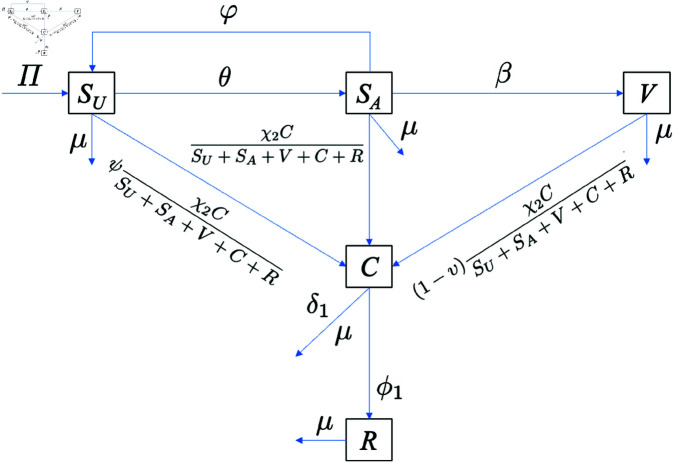
COVID-19-only transmission diagram. Transmission diagram of COVID-19-only model in system (10).

The specific case of model (3), which considers a population affected solely by COVID-19, is represented by system (10) below. The corresponding transmission diagram is shown in Fig 3.

$$S_{U}^{\prime }=\Pi -\psi \frac{\chi_{2}C}{S_{U}+S_{A}+V+C+R}S_{U}-\mu S_{U}-\theta S_{U}+\varphi S_{A},\;S_{A}^{\prime }=\theta S_{U}-\frac{\chi_{2}C}{S_{U}+S_{A}+V+C+R}S_{A}-(\mu +\varphi +\beta )S_{A},\;V^{\prime }=\beta S_{A}-(1-\nu )\frac{\chi_{2}C}{S_{U}+S_{A}+V+C+R}V-\mu V,\;C^{\prime }=\frac{\chi_{2}C}{S_{U}+S_{A}+V+C+R}\left(\psi S_{U}+S_{A}+(1-\nu )V\right)-(\phi_{1}+\mu +\delta_{1})C,\;R^{\prime }=\phi_{1}C-\mu R.$$ (10)

In a similar manner to that presented in Lemma 1, the following lemma can be similarly derived.

**Lemma 3.** *If the initial conditions of system (10) satisfy $S_{U}(0)>0,S_{A}(0)\geq 0,V(0)\geq 0,C(0)\geq 0,$ and R(0)≥0, then the solutions $S_{U}(t),S_{A}(t),V(t),C(t),$ and R(t) remain non-negative for all t > 0.*

The next lemma discusses the feasible region of the COVID-19 model in Eq 10. This theorem can be proved using a similar approach to that used in Lemma 2.

**Lemma 4.** *The feasible region defined by*


$$\Omega_{COV}=\left\{(S_{U},S_{A},V,C,R)\in {\mathbb{R}}_{+}^{5}:S_{U}+S_{A}+V+C+R\leq \left\{N(0),\frac{\Pi }{\mu }\right\}\right\},$$

*is positively invariant and attracting with regard of system (10).*

#### 3.2.1 COVID-19-free equilibrium and the basic reproduction number ${ℛ}_{0_{COV}}$.

In this section, we calculate the COVID-19-free equilibrium and the basic reproduction number corresponding to the COVID-19 model in Eq 10. By setting the right-hand side of system (10) to zero and assuming *C* = 0 and *R* = 0, the COVID-19-free equilibrium is obtained as follows:

$${ℰ}_{COV}^{0}=(S_{U}^{0},S_{A}^{0},V^{0},C^{0},R^{0}),$$ (11)

with $S_{U}^{0}=\frac{\Pi (\;\mu +\varphi +\beta )\;}{(\;\theta +\mu )\;(\;\mu +\varphi +\beta )\;-\varphi \theta }$, $S_{A}^{0}=\frac{\Pi \theta }{(\;\theta +\mu )\;(\;\mu +\varphi +\beta )\;-\varphi \theta },$ $V^{0}=\frac{\Pi \beta \theta }{\mu [(\;\theta +\mu )\;(\;\mu +\varphi +\beta )\;-\varphi \theta ]}$, *C*^0^ = 0, and *R*^0^ = 0. We apply a similar method as in the previous section to determine the basic reproduction number of the COVID-19 model in (10), denoted by ${ℛ}_{0_{COV}}$. Through direct calculation, we obtain:

$${ℛ}_{0_{COV}}=\frac{\chi_{2}(\psi \mu (\mu +\varphi +\beta )+\theta \mu +(1-\vartheta )\beta \theta )}{\left(\phi_{1}+\mu +\delta_{1})[\mu (\mu +\varphi +\beta )+\theta \mu +\beta \theta \right]}.$$ (12)

#### 3.2.2 The COVID-19 endemic equilibrium point.

Let $z=\frac{\chi_{2}C^{*}}{N^{*}}$ represent the infection term in the steady-state condition. Hence, Eq 10 in its steady-state condition can be expressed as follows:


$$\Pi -\psi zS_{U}^{*}-\mu S_{U}^{*}-\theta S_{U}^{*}+\varphi S_{A}^{*}=0,$$

$$\theta S_{U}^{*}-zS_{A}^{*}-\mu S_{A}^{*}-\varphi S_{A}^{*}-\beta S_{A}^{*}=0,$$

$$\beta S_{A}^{*}-(1-\vartheta )zV^{*}-\mu V^{*}=0,$$

$$\psi zS_{U}^{*}+zS_{A}^{*}+(1-\vartheta )zV^{*}-\phi_{1}C^{*}-(\mu +\delta_{1})C^{*}=0,$$

$$\phi_{1}C^{*}-\mu R^{*}=0.$$

Hence, the COVID-19 endemic equilibrium is given by:

$${ℰ}_{COV}^{*}=(\;S_{U}^{*},S_{A}^{*},V^{*},C^{*},R^{*})\;,$$ (13)

where

$$=S_{U}^{*}=\frac{\Pi +\varphi S_{A}^{*}}{\psi z+\mu +\theta },\;\;\;\;\;\;S_{A}^{*}=\frac{\theta S_{U}^{*}}{z+\rho },\;\;\;\;\;\;\;V^{*}=\frac{\theta \beta S_{U}^{*}}{\left(\epsilon z+\mu )(z+\rho \right)},\;C^{*}=\frac{\psi zS_{U}^{*}+zS_{A}^{*}+\epsilon zV^{*}}{d+\delta_{1}},\;\;\;\;\;\;R^{*}=\frac{\phi_{1}}{\mu }C^{*},$$ (14)

with ρ=μ+φ+β, $d=\phi_{1}+\mu$, and ϵ=1−ϑ. Substitute above equation in (??) to $z=\frac{\chi_{2}C^{*}}{N^{*}}$ give:

$$\mu (d+\delta_{1})\left(S_{U}^{*}+S_{A}^{*}+V^{*}\right)=(\mu \chi_{2}-zd)(\psi S_{U}^{*}+S_{A}^{*}+\epsilon V^{*}).$$ (15)

Hence, substituting $S_{A}^{*}$ and ${\text{V}}^{*}$ to (??) yields the following polynomial for determining the existence of a solution for *z*:

$$P(z)=a_{3}z^{3}+a_{2}z^{2}+a_{1}z+a_{0}=0,$$ (16)

where


$$a_{3}=\epsilon \psi d,$$

$$a_{2}=d(\epsilon \psi \rho +\epsilon \theta +\mu \psi )+\epsilon \mu (-\psi \chi_{2}+d+\delta_{1}),$$

$$a_{1}=-\mu \chi_{2}(\epsilon \psi \rho +\psi \mu +\epsilon \theta )+d(\beta \epsilon \theta +\mu \psi \rho +\theta \mu )+\mu (d+\delta_{1})(\epsilon \rho +\epsilon \theta +\mu ),$$

$$a_{0}=\mu (d+\delta_{1})(\mu \rho +\mu \theta +\beta \theta )\left(1-{ℛ}_{0_{COV}}\right).$$

The polynomial *P*(*z*) in (??) is a cubic polynomial in *z*. According to the Fundamental Theorem of Algebra, it will always have a maximum of three roots. Furthermore, it is evident that since *a*_3_>0 and $a_{0}<0↔{ℛ}_{0_{COV}}>1$, the polynomial *P*(*z*) will always have at least one positive root for *z* if ℛ0COV>1. Thus, we arrive at the following lemma.

**Lemma 5.** *The COVID-19-only model in (10) will always have at least one endemic equilibrium if ${ℛ}_{0_{COV}}>1$.*

To determine the possible number of positive roots of *P*(*z*), we apply Descartes’ rule of signs, and the results are summarized in Table 2. It is clear that if ${ℛ}_{0_{COV}}>1$, it is always possible to have an endemic equilibrium ${ℰ}_{COV}^{*}$, either one or three. On the other hand, if ${ℛ}_{0_{COV}}<1$, while it is possible to have no endemic equilibrium ${ℰ}_{COV}^{*}$, it is also possible to have two ${ℰ}_{COV}^{*}$ for ${ℛ}_{0_{COV}}<1$ (refer to rows 3, 5, and 7 of Table 2).

**Table 2:** Descartes rules of sign for polynomial *P*(*z*).

$a_{3}$	$a_{2}$	$a_{1}$	$a_{0}$	${ℛ}_{0_{COV}}$	Possible number of positive roots
+	+	+	+	<1	0
+	+	+	–	>1	1
+	+	–	+	<1	2, 0
+	+	–	–	>1	1
+	–	+	+	<1	2, 0
+	–	+	–	>1	3, 1
+	–	–	+	<1	2, 0
+	–	–	–	>1	1

#### 3.2.3 The stability of COVID-19-free equilibrium point.

We apply the linearization technique to analyze the stability of system (10) around $ℰ{COV}^{0}$. By evaluating the Jacobian matrix of system (10) at $ℰ{COV}^{0}$, we obtain:


$$J|({ℰ}_{COV}^{0})=[\begin{array}{ccccc} -\mu -\theta & \varphi & 0 & -\frac{\psi \chi_{2}\mu (\mu +\varphi +\beta )}{\mu (\mu +\varphi +\beta )+\mu \theta +\beta \theta } & 0\\ \theta & -(\mu +\varphi +\beta ) & 0 & -\frac{\chi_{2}\mu \theta }{\mu (\mu +\varphi +\beta )+\mu \theta +\beta \theta } & 0\\ 0 & \beta & -\mu & -\frac{(1-\vartheta )\chi_{2}\beta \theta }{\mu (\mu +\varphi +\beta )+\mu \theta +\beta \theta } & 0\\ 0 & 0 & 0 & ℛ-(\phi_{1}+\mu +\delta_{1}) & 0\\ 0 & 0 & 0 & \phi_{1} & -\mu \end{array}],$$

where $ℛ=\frac{\chi_{2}(\psi \mu (\mu +\varphi +\beta )+\theta \mu +(1-\vartheta )\beta \theta )}{\mu (\mu +\varphi +\beta )+\theta \mu +\beta \theta }$.

Three explicit eigenvalues are obtained: $\lambda_{1}=\lambda_{2}=-\mu$ and $\lambda_{3}=(\phi_{1}+\mu +\delta_{1})\left({ℛ}_{0_{COV}}-1\right)$. It is evident that $\lambda_{1}$ and $\lambda_{2}$ are always negative, while $\lambda_{3}$ is negative if and only if ${ℛ}_{0_{COV}}<1$. The remaining two eigenvalues are determined from the roots of the following polynomial:

$$\lambda^{2}+(2\mu +\theta +\varphi +\beta )\lambda +\mu^{2}+\mu \varphi +\mu \beta +\theta \mu +\theta \beta =0.$$ (17)

Since the coefficients of the above polynomial are always positive, the roots will always have negative real parts, regardless of any conditions. Thus, we can state the following theorem.

**Theorem 4.** *The COVID-19 free equilibrium ${ℰ}_{COV}^{0}$ of COVID-19 only model in (10) is always locally asymptotically stable if ${ℛ}_{0_{COV}}<1$ and unstable if ${ℛ}_{0_{COV}}>1$.*

#### 3.2.4 The stability of COVID-19 endemic equilibrium point.

We use the center-manifold approach around ℛ0COV=1 to analyze the local stability of the COVID-19 endemic equilibrium in this section. This method was first introduced by Castillo and Song in [53]. To apply this method, we rewrite the model variables as follows:


$$S_{U}=x_{1},\;S_{A}=x_{2},\;V=x_{3},\;C=x_{4},\;\text{dan}\;R=x_{5},$$

with $N=x_{1}+x_{2}+x_{3}+x_{4}+x_{5}$. Hence, the COVID-19 model in (10) now reads as follows:

$$g_{1}=\Pi -\psi \frac{\chi_{2}x_{4}}{N}x_{1}-\mu x_{1}-\theta x_{1}+\varphi x_{2},$$ (18a)

$$g_{2}=\theta x_{1}-\frac{\chi_{2}x_{4}}{N}x_{2}-\mu x_{2}-\varphi x_{2}-\beta x_{2},$$ (18b)

$$g_{3}=\beta x_{2}-(1-\vartheta )\frac{\chi_{2}x_{4}}{N}x_{3}-\mu x_{3},$$ (18c)

$$g_{4}=\psi \frac{\chi_{2}x_{4}}{N}x_{1}+\frac{\chi_{2}x_{4}}{N}x_{2}+(1-\vartheta )\frac{\chi_{2}x_{4}}{N}x_{3}-\phi_{1}x_{4}-(\mu +\delta_{1})x_{4},$$ (18d)

$$g_{5}=\phi_{1}x_{4}-\mu x_{5}.$$ (18e)

Next, we choose $\chi_{2}$ as the bifurcation parameter. By solving ${ℛ}_{0_{COV}}=1$ with respect to $\chi_{2}$, we obtain:

$$\chi_{2}^{*}=\frac{\left(\phi_{1}+\mu +\delta_{1})[\mu (\mu +\varphi +\beta )+\theta \mu +\beta \theta \right]}{\left(\psi \mu (\mu +\varphi +\beta )+\theta \mu +(1-\vartheta )\beta \theta \right)}.$$ (19)

Linearizing system (18) at $\chi_{2}=\chi_{2}^{*}$ and at COVID-19 free equilibrium point yields:


$$𝐉({ℰ}_{COV}^{0},\chi_{2}^{*})=[\begin{array}{ccccc} -(\mu +\theta ) & \varphi & 0 & j_{1} & 0\\ \theta & -(\beta +\mu +\varphi ) & 0 & j_{2} & 0\\ 0 & \beta & -\mu & j_{3} & 0\\ 0 & 0 & 0 & 0 & 0\\ 0 & 0 & 0 & \phi_{1} & -\mu \end{array}],$$

with $j_{1}=-\frac{\psi \mu (\phi_{1}+\mu +\delta_{1})}{\left(\psi \mu (\mu +\varphi +\beta )+\theta \mu +(1-\vartheta )\beta \theta \right)}$, $j_{2}=-\frac{\mu \theta (\phi_{1}+\mu +\delta_{1})}{\left(\psi \mu (\mu +\varphi +\beta )+\theta \mu +(1-\vartheta )\beta \theta \right)}$, and $j_{3}=-\frac{\left(1-\vartheta )\beta \theta (\phi_{1}+\mu +\delta_{1}\right)}{\left(\psi \mu (\mu +\varphi +\beta )+\theta \mu +(1-\vartheta )\beta \theta \right)}$.

From direct calculation, we find a simple zero eigenvalue of $𝐉({ℰ}_{COV}^{0},\chi_{2}^{*})$. Two explicit eigenvalues are negative, namely $\lambda_{2}=\lambda_{3}=-\mu$, while the remaining two eigenvalues are determined from the positive roots of the following polynomial:


$$\left(\lambda^{2}+(2\mu +\theta +\varphi +\beta )\lambda +\mu^{2}+\mu \varphi +\mu \beta +\theta \mu +\theta \beta \right)=0.$$

Since the coefficients of the above polynomial are all positive, then $\lambda_{3}$ and $\lambda_{4}$ always have negative real parts. Given that there is a simple zero eigenvalue while the other four eigenvalues have a negative real part, we can apply the Castillo-Song bifurcation theorem to analyze the behavior of the system near the branching point at ${ℛ}_{0_{COV}}=1$.

To determine the type of bifurcation near ${ℛ}_{0_{COV}}=1$,we need to evaluate the sign of the following indicator as specified by the Castillo-Song theorem:

$$A=\sum_{k,i,j=1}^{5}v_{k}w_{i}w_{j}\frac{\partial^{2}g_{k}}{\partial x_{i}\partial x_{j}}({ℰ}_{COV}^{0}),\;\;\;\;\;\;B=\sum_{k,i=1}^{5}v_{k}w_{i}\frac{\partial^{2}g_{k}}{\partial x_{i}\partial \chi_{2}}({ℰ}_{COV}^{0}),$$ (20)

where *w*_*k*_ and $v_{k}$ are the right and left eigenvectors of $𝐉({ℰ}_{COV}^{0},\chi_{2}^{*})$ correspond to the zero eigenvalue.

From direct calculation, the right eigenvector of $𝐉({ℰ}_{COV}^{0},\chi_{2}^{*})$ correspond λ=0 is given by:


$$𝐰=[w_{1}\;\;\;w_{2}\;\;\;w_{3}\;\;\;w_{4}\;\;\;w_{5}],$$

where


$$w_{1}=\frac{w_{2}(\psi (\beta +\mu +\varphi )+\theta \varphi )}{\theta (\psi (\mu +\beta +\varphi )+\mu +\theta )},$$

$$w_{2}=w_{2},$$

$$w_{3}=\frac{w_{2}\beta \left[\;(1-\vartheta )\left\{\mu (\mu +\beta +\varphi )+\mu \theta +\beta \theta \right\}+\psi \mu (\mu +\beta +\varphi )+\mu (\mu +\theta )\right]\;}{\mu^{2}(\psi (\mu +\beta +\varphi )+\mu +\theta )},$$

$$w_{4}=-\frac{w_{2}\left[\;\mu (\mu +\beta +\varphi )+\mu \theta +\beta \theta \right]\;\left[\;\psi \mu (\mu +\beta +\varphi )+\mu \theta +(1-\vartheta )\theta \beta \right]\;}{\theta \mu (\psi (\mu +\beta +\varphi )+\mu +\theta )(\phi_{1}+\mu +\delta_{1})},$$

$$w_{5}=-\frac{w_{2}\phi_{1}\left[\;\mu (\mu +\beta +\varphi )+\mu \theta +\beta \theta \right]\;\left[\;\psi \mu (\mu +\beta +\varphi )+\mu \theta +(1-\vartheta )\theta \beta \right]\;}{\theta \mu^{2}(\psi (\mu +\beta +\varphi )+\mu +\theta )(\phi_{1}+\mu +\delta_{1})}.$$

Furthermore, the left eigenvector corresponds to λ=0 is given by


$$𝐯=[v_{1}\;\;\;v_{2}\;\;\;v_{3}\;\;\;v_{4}\;\;\;v_{5}]=[0\;\;\;0\;\;\;0\;\;\;1\;\;\;0].$$

With the left and right eigenvectors at hand, we can directly compute the bifurcation indicators A and B. From direct calculation, we obtain:


$$A=\sum_{k,i,j=1}^{n}v_{k}w_{i}w_{j}\frac{\partial^{2}g_{k}}{\partial x_{i}\partial x_{j}}({ℰ}_{COV}^{0}),$$

$$=-\frac{2\chi_{2}^{*}(\mu (\mu +\beta +\varphi )+\mu \theta +\beta \theta )P_{1}P_{2}}{\Pi \mu^{2}\theta^{2}(\phi_{1}+\mu +\delta_{1}{)}^{2}(\psi (\mu +\beta +\varphi )+\mu +\theta {)}^{2}},$$

where


$$P_{1}=\psi \mu (\mu +\beta +\varphi )+\mu \theta +(1-\vartheta )\beta \theta ,\;P_{2}=\beta^{2}(1-\vartheta {)}^{2}\mu^{2}\theta +\beta^{2}(1-\vartheta {)}^{2}\mu \theta^{2}+\beta^{2}(1-\vartheta {)}^{2}\mu \theta \delta_{1}+\beta^{2}(1-\vartheta {)}^{2}\mu \theta \phi_{1}+\mu^{4}\theta \;+\beta^{2}(1-\vartheta {)}^{2}\theta^{2}\phi_{1}+\beta^{2}(1-\vartheta )\mu^{2}\psi \theta -\beta^{2}(1-\vartheta )\mu \psi \theta \delta_{1}+\beta^{2}(1-\vartheta )\mu \psi \theta \phi_{1}\;+\beta^{2}\mu^{3}\psi^{2}+\beta^{2}\mu^{2}\psi^{2}\phi_{1}+\beta (1-\vartheta {)}^{2}\mu^{3}\theta +\beta (1-\vartheta {)}^{2}\mu^{2}\theta^{2}+\beta (1-\vartheta {)}^{2}\mu^{2}\theta \varphi \;+\beta (1-\vartheta {)}^{2}\mu^{2}\theta \delta_{1}+\beta (1-\vartheta {)}^{2}\mu^{2}\theta \phi_{1}+\beta \epsilon^{2}\mu \theta^{2}\delta_{1}+\beta (1-\vartheta {)}^{2}\mu \theta^{2}\phi_{1}+\mu^{3}\theta^{2}\;+\beta (1-\vartheta {)}^{2}\mu \theta \varphi \delta_{1}+\beta \epsilon^{2}\mu \theta \varphi \phi_{1}+\beta \epsilon \mu^{3}\psi \theta +\beta \epsilon \mu^{2}\psi \theta \varphi -\beta (1-\vartheta )\mu^{2}\psi \theta \delta_{1}\;+\beta (1-\vartheta )\mu^{2}\psi \theta \phi_{1}-\beta (1-\vartheta )\mu \psi \theta \varphi \delta_{1}+\beta (1-\vartheta )\mu \psi \theta \varphi \phi_{1}+\beta (1-\vartheta )\mu^{3}\theta \;+2\beta \mu^{3}\psi^{2}\varphi +2\beta \mu^{3}\psi^{2}\phi_{1}+2\beta \mu^{2}\psi^{2}\varphi \phi_{1}+\mu^{5}\psi^{2}+\mu^{4}\psi^{2}\phi_{1}+\beta (1-\vartheta )\mu \theta^{2}\phi_{1}\;+\mu^{3}\psi^{2}\varphi^{2}+2\mu^{3}\psi^{2}\varphi \phi_{1}+\mu^{2}\psi^{2}\varphi^{2}\phi_{1}+\beta (1-\vartheta )\mu^{2}\theta^{2}+\mu^{3}\theta \delta_{1}+2\beta \mu^{4}\psi^{2}\;+\beta (1-\vartheta )\mu^{2}\theta \delta_{1}+\beta (1-\vartheta )\mu^{2}\theta \phi_{1}-\beta (1-\vartheta )\mu \theta^{2}\delta_{1}+2\mu^{4}\psi^{2}\varphi +\mu^{2}\theta^{2}\phi_{1}\;+\beta \mu^{3}\psi \theta -\beta \mu^{2}\psi \theta \delta_{1}+\beta \mu^{2}\psi \theta \phi_{1}+2\mu^{3}\psi \theta \varphi -\mu^{3}\psi \theta \delta_{1}+\mu^{3}\psi \theta \phi_{1}+\mu^{4}\psi \theta \;+2\mu^{2}\psi \theta \varphi \phi_{1}+\mu^{3}\theta \phi_{1}.$$

Furthermore,


$$B=\sum_{k,i=1}^{n}v_{k}w_{i}\frac{\partial^{2}g_{k}}{\partial x_{i}\partial \chi_{2}}({ℰ}_{COV}^{0}),$$

$$=\frac{{\left(\psi \mu (\mu +\beta +\varphi )+\mu \theta +(1-\vartheta )\beta \theta \right)}^{2}}{\mu \theta (\mu (\mu +\beta +\varphi )+\mu \theta +\beta \theta )(\phi_{1}+\mu +\delta_{1})}.$$

From the above calculation, it is evident that B is always positive, while A can be either positive or negative, depending on the sign of *P*_2_. Note that *P*_2_ can be rewritten in the following form:

$$P_{2}=Q_{1}\left(\frac{Q_{2}}{Q_{1}}-1\right),$$ (21)

where


$$Q_{1}=\mu \theta \delta_{1}[\beta^{2}(1-\vartheta )\psi +\beta (1-\vartheta )\mu \psi +\beta (1-\vartheta )\psi \varphi +\beta (1-\vartheta )\theta +\beta \mu \psi +\mu^{2}\psi ],\;Q_{2}=\mu \theta \phi_{1}[\beta^{2}(1-\vartheta )\psi +\beta (1-\vartheta )\mu \psi +\beta (1-\vartheta )\psi \varphi +\beta (1-\vartheta )\theta +\beta \mu \psi +\mu^{2}\psi ]\;+\mu^{2}\theta [\beta^{2}(1-\vartheta )\psi +\beta (1-\vartheta )\mu \psi +\beta (1-\vartheta )\psi \varphi +\beta (1-\vartheta )\theta +\beta \mu \psi +\mu^{2}\psi ]\;+\beta^{2}(1-\vartheta {)}^{2}\mu^{2}\theta +\beta^{2}(1-\vartheta {)}^{2}\mu \theta^{2}+\beta^{2}(1-\vartheta {)}^{2}\mu \theta \delta_{1}+\beta^{2}(1-\vartheta {)}^{2}\mu \theta \phi_{1}\;+\beta^{2}(1-\vartheta {)}^{2}\theta^{2}\phi_{1}+\beta^{2}\mu^{3}\psi^{2}+\beta^{2}\mu^{2}\psi^{2}\phi_{1}+\beta (1-\vartheta {)}^{2}\mu^{3}\theta +\beta (1-\vartheta {)}^{2}\mu^{2}\theta^{2}\;+\beta (1-\vartheta {)}^{2}\mu^{2}\theta \varphi +\beta (1-\vartheta {)}^{2}\mu^{2}\theta \delta_{1}+\beta (1-\vartheta {)}^{2}\mu^{2}\theta \phi_{1}+\beta (1-\vartheta {)}^{2}\mu \theta^{2}\delta_{1}\;+\beta (1-\vartheta {)}^{2}\mu \theta^{2}\phi_{1}+\beta (1-\vartheta {)}^{2}\mu \theta \varphi \delta_{1}+\beta (1-\vartheta {)}^{2}\mu \theta \varphi \phi_{1}+2\beta \mu^{4}\psi^{2}+\mu^{3}\theta^{2}\;+2\beta \mu^{3}\psi^{2}\phi_{1}+2\beta \mu^{2}\psi^{2}\varphi \phi_{1}+\mu^{5}\psi^{2}+2\mu^{4}\psi^{2}\varphi +2\beta \mu^{3}\psi^{2}\varphi +\mu^{3}\psi^{2}\varphi^{2}+\mu^{4}\theta \;+\mu^{2}\psi^{2}\varphi^{2}\phi_{1}+\beta (1-\vartheta )\mu^{3}\theta +\beta (1-\vartheta )\mu^{2}\theta \delta_{1}+\beta (1-\vartheta )\mu^{2}\theta \phi_{1}+2\mu^{3}\psi \theta \varphi \;+2\mu^{2}\psi \theta \varphi \phi_{1}+\mu^{3}\theta \phi_{1}+\mu^{3}\theta \delta_{1}+\mu^{2}\theta^{2}\phi_{1}+2\mu^{3}\psi^{2}\varphi \phi_{1}+\mu^{4}\psi^{2}\phi_{1}.$$

Let ${ℛ}_{s}=\frac{Q_{2}}{Q_{1}}$. Then, we have $A>0\Leftrightarrow {ℛ}_{s}<1$ and vice versa. Since B is always positive, the following theorem describes the type of bifurcation of our COVID-19-only model in (10) at ℛ0COV=1.

**Theorem 5.** *The COVID-19 only model in (10) will:*

1. *undergoes a forward bifurcation at ${ℛ}_{0_{COV}}=1$ if ${ℛ}_{s}>1$, and*
2. *undergoes a backward bifurcation at ${ℛ}_{0_{COV}}=1$ if ${ℛ}_{s}<1$.*

From Theorem 5 and the existence criteria of the COVID-19 equilibrium in Table 2, we can conclude that the COVID-19-only model in (10):

1. always have at least one stable endemic equilibrium for ${ℛ}_{0_{COV}}>1$ but close to one if ${ℛ}_{s}>1$,
2. always has two endemic equilibria for certain values of ${ℛ}_{0_{COV}}<1$ if ${ℛ}_{s}<1$, where the smaller equilibrium is ununstable nd the larger one is stable.

### 3.3 COVID-19 and HIV co–infection model

In this final subsection, we analyze the co–infection model of HIV and COVID-19 presented in Eq 3. The analysis includes the criteria for the positiveness of solutions and the calculation of the reproduction number.

Using a similar approach to that applied in the HIV-only and COVID-19-only models, the positiveness criteria for the solutions of the co–infection model between HIV and COVID-19 are provided in the following theorem.

**Lemma 6.** *If the initial conditions of system (3) given by $S_{U}(0)>0,S_{A}(0)\geq 0,V(0)\geq 0,C\geq 0,R\geq 0,H(0)\geq 0,A\geq 0,C_{H}\geq 0,C_{A}\geq 0,$ then the solutions $S_{U}(t),S_{A}(t),V(t),C(t),R(t)$, H(t),A(t),C_H_(t) and C_A_(t) are non-negative for all time t > 0.*

The disease-free equilibrium of the co–infection model in (3) is given by:

$${ℰ}^{0}=\left(S_{U}^{0},S_{A}^{0},V^{0},C^{0},R^{0},H^{0},A^{0},C_{H}^{0},C_{A}^{0}\right)=\left(S_{U}^{0},S_{A}^{0},V^{0},0,0,0,0,0,0\right),$$ (22)

with $S_{U}^{0}=\frac{\Pi (\;\mu +\varphi +\beta )\;}{(\;\theta +\mu )\;(\;\mu +\varphi +\beta )\;-\varphi \theta }$, $S_{A}^{0}=\frac{\Pi \theta }{(\;\theta +\mu )\;(\;\mu +\varphi +\beta )\;-\varphi \theta },$ and $V^{0}=\frac{\Pi \beta \theta }{\mu [(\theta +\mu )(\mu +\varphi +\beta )-\varphi \theta ]}.$ Using the next-generation method [53], the basic reproduction number of the co–infection model is given by:

$${ℛ}_{0}=\max\left\{{ℛ}_{0_{HIV}},{ℛ}_{0_{COV}}\right\},$$ (23)

where ${ℛ}_{0_{COV}}=\frac{\chi_{2}(\psi \mu (\mu +\varphi +\beta )+\theta \mu +(1-\vartheta )\beta \theta )}{\left(\phi_{1}+\mu +\delta_{1})[\mu (\mu +\varphi +\beta )+\theta \mu +\beta \theta \right]}$ and ${ℛ}_{0_{HIV}}=\frac{\chi_{1}(\omega \gamma_{1}+\mu +\delta_{2})}{\left(\gamma_{1}+\mu )(\delta_{2}+\mu \right)}.$

More discussion of the dynamical properties of the COVID-19 and HIV co–infection model will be discussed in the following section.

## 4 Numerical study of the COVID-HIV co-infection model

This section will present a detailed numerical investigation of the dynamical behavior of the HIV-COVID co-infection model (3) introduced earlier, when key parameters of the model are perturbed. This study will be performed employing path-following (continuation) methods implemented using the continuation software COCO [54]. This is a numerical platform based on MATLAB designed to solve continuation problems covering, in a broad extent, the analysis and bifurcation detection routines available in classical continuation tools, such as AUTO [55] and MATCONT [56]. In particular, in this work we will use extensively the capabilities for continuation and bifurcation analysis of branches of equilibria in smooth systems of odes, with special emphasis on the detection of codimension-1 and -2 phenomena.

**Fig 4:**
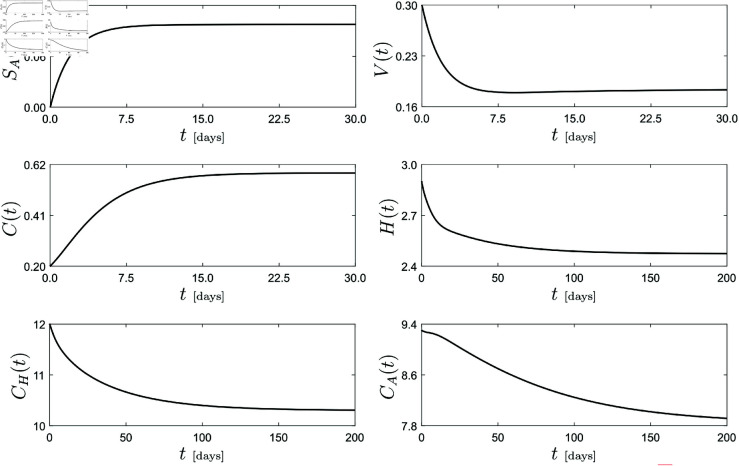
Dynamical response of the HIV-COVID co-infection model (3). Solution was computed for the parameter values given in Table 1, with $\chi_{2}=0.4$, ω=1.1, η=0.5, ψ=1.2, θ=0.1, φ=0.1, β=0.4 and $\Lambda_{1}=\Lambda_{2}=2$.

To start our study, we will set first the parameter values of the HIV-COVID co-infection model (3) according to Table 1, considering $\chi_{2}=0.4$, ω=1.1, η=0.5, ψ=1.2, θ=0.1, φ=0.1, β=0.4 and $\Lambda_{1}=\Lambda_{2}=2$, which fall within the defined realistic parameter ranges. Under this parameter scenario, the dynamical response of system (3) obtained via direct numerical integration is presented in Fig 4. This diagram shows the time response for selected compartments of the model: *S*_*A*_, *V*, *C*, *H*, *C*_*H*_ and *C*_*A*_. The observed behavior reveals an evolution that after transients approaches a full endemic equilibrium, that is, a steady state where both diseases COVID and HIV are present in the system. This equilibrium will be the starting point of our numerical investigation, where one of the main questions concerns how this undesirable steady state is perturbed when key system parameters vary and how to bring this state to a disease-free scenario.

**Fig 5:**
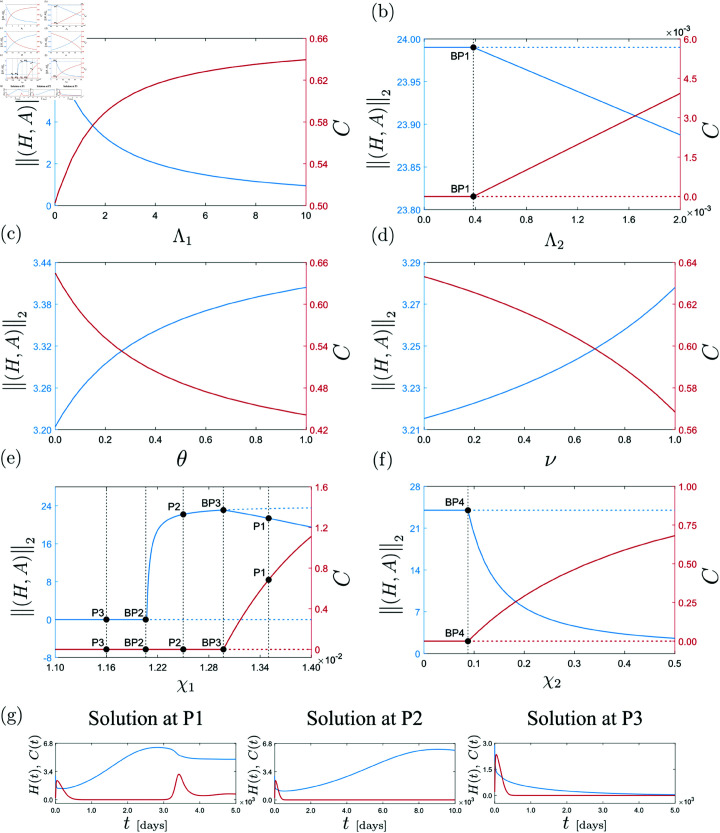
Continuation of parameter-dependent equilibria of model (3). This experiment conducted with respect to $\Lambda_{1}$, $\Lambda_{2}$, θ, ν, $\chi_{1}$ and $\chi_{2}$, calculated for the parameter values used in Fig 4. Panels (a)–(f) show in the vertical axis on the left (blue) the variations of the HIV-related compartments of the model. The vertical axis on the right (red) displays the parameter-dependence of the COVID compartment *C*. In the bifurcation pictures, stable and unstable equilibria are represented by solid and dashed lines, respectively. The bifurcation analysis reveals the presence of several branching points listed as follows: BP1 ($\Lambda_{2}\approx 3.8405\;\times \;10^{-4}$), BP2 ($\chi_{1}\approx 0.01206$), BP3 ($\chi_{1}\approx 0.01297$) and BP4 ($\chi_{2}\approx 0.08761$). Panel (g) shows time responses of system (3) computed at the test points P1 ($\chi_{1}=0.0135$), P2 ($\chi_{1}=0.0125$) and P3 ($\chi_{1}=0.0116$). In these plots, the red series corresponds to *C*(*t*) while the blue series to *H*(*t*). In what follows, this color code will be employed to present time plots of the *H* and *C* compartments in the same graph.

Next, we will use numerical continuation (path-following) methods to study how this full endemic steady state is affected when selected system parameters are perturbed. The result of this continuation analysis is presented in Fig 5, panels (a)–(f). In this picture, the horizontal axis displays the selected bifurcation parameter, while the vertical axis on the left (in blue) shows the variations of the Euclidean norm of the HIV-related compartments (*H*,*A*), which gives a measure of the intensity of the disease in the model. Moreover, the vertical axis on the right (in red) presents the response of the COVID compartment *C*.

The numerical continuation of the full endemic equilibrium subject to variations to the COVID transmission factor for HIV patients $\Lambda_{1}$ is presented in Fig 5(a). As can be observed, an increment of this parameter produces a growth of the COVID compartment, while the HIV-related components decrease. This is due to the fact that larger $\Lambda_{1}$ induces a larger transmission rate of COVID, according to the second term of the *C*_*H*_-component of system (3). For this reason, the individuals with only HIV are transferred with a larger rate to the co-infection compartment *C*_*H*_, due to which the HIV-related compartments decrease. An analogous scenario is encountered when the COVID transmission factor for AIDS patients $\Lambda_{2}$ is varied, see Fig 5(b). Under the same reasoning, the COVID presence in the system increases, while the number of patients with HIV or AIDS only reduces as $\Lambda_{2}$ grows. In this case, however, a critical point BP1 ($\Lambda_{2}\approx 3.8405\;\times \;10^{-4}$) is found, corresponding to a branching. Here, the stable endemic steady state turns into a stable COVID-free equilibrium with HIV in the system.

Fig 5(c) presents the continuation of the full endemic steady state considering variations of the COVID awareness campaign rate θ. Recall that higher θ means increased protection against COVID through vaccination and use of protection equipment such as face masks. Therefore, is is expected to observe a reduction in the COVID compartment as θ increases, see Fig 5(c). At the same time, the compartments related to HIV-COVID co-infection reduce, due to which the population with HIV only increases. An analogous situation is encountered when the COVID vaccine efficacy is perturbed, see panel (d). The COVID infection rate $\chi_{2}$ is studied in Fig 5(f), showing an increment of the COVID compartment as $\chi_{2}$ grows. This increases the HIV-COVID co-infections, hence producing a reduction of the population with HIV only. In this diagram, another branching bifurcation is found, this time at BP4 ($\chi_{2}\approx 0.08761$). Here, a stable full endemic equilibrium becomes a stable endemic equilibrium with HIV and no COVID. A remarkably different situation is encountered when the HIV infection rate $\chi_{1}$ is perturbed, see Fig 5(e). A full endemic equilibrium is encountered for large $\chi_{1}$, as expected. When this parameter decreases, a critical point BP3 ($\chi_{1}\approx 0.01297$) is found, where the COVID compartment becomes zero and only HIV survives in the system. This means that, for the considered biological scenario, the presence of COVID requires a sufficiently high transmissibility of the human immunodeficiency virus. For a different parameter setting, of course, this needs not be the case, as will be seen later. After COVID disappears, a further reduction of $\chi_{1}$ produces a decrement of HIV until it vanishes from the system at the branching point BP2 ($\chi_{1}\approx 0.01206$). Below this point, the disease-free equilibrium gains stability, where none of the diseases are present in the system, a situation that could not be observed by varying the previous parameters.

**Fig 6:**
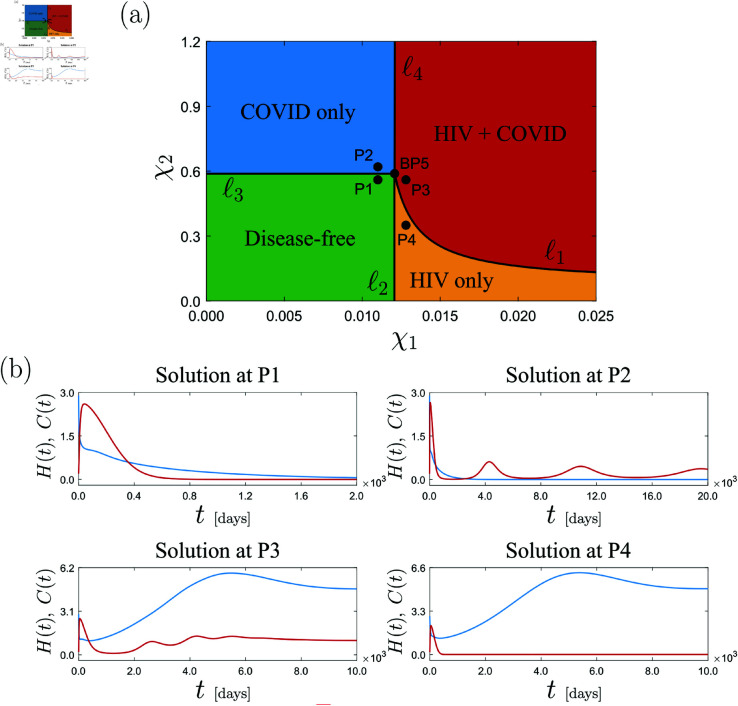
Codimension-2 of model (3). (a) Continuation of the bifurcation points found in Fig 5(e) with respect to the parameters $\chi_{1}$ and $\chi_{2}$. The diagram shows the continuation of the bifurcations BP3 ($\ell_{1}$) and BP2 ($\ell_{2}$), which intersect each other at the point BP5 ($\chi_{1}\approx 0.01206$, $\chi_{2}\approx 0.5886$). From this point, two further curves of branching points emanate, labeled $\ell_{3}$ and $\ell_{4}$. The resulting curves divides the parameter space into four regions as indicated in the diagram. Panel (b) shows time plots of system (3) calculated at the selected points P1 ($\chi_{1}=0.011$, $\chi_{2}=0.56$), P2 ($\chi_{1}=0.011$, $\chi_{2}=0.62$), P3 ($\chi_{1}=0.0128$, $\chi_{2}=0.56$) and P4 ($\chi_{1}=0.0128$, $\chi_{2}=0.35$).

To conclude this section, we will now carry out a two-parameter study of the critical points encountered above. Specifically, we will perform a numerical continuation of the codimension-1 points BP3 and BP2 with respect to the HIV and COVID infection rates $\chi_{1}$ and $\chi_{2}$, respectively. The outcome of this study is displayed in Fig 6(a). This picture presents the continuation of the bifurcations BP3 ($\ell_{1}$) and BP2 ($\ell_{2}$) with respect two parameters, where the resulting curves intersect each other at the point BP5 ($\chi_{1}\approx 0.01206$, $\chi_{2}\approx 0.5886$). Here, a degenerate codimension-2 branching phenomenon takes place, where the HIV and COVID endemic branches become zero simultaneously. This point is characterized by the condition ${ℛ}_{0_{HIV}}={ℛ}_{0_{COV}}=1$ (see (6) and (??)), which can be achieved, in a generic manner, by varying two parameters (hence codimension-2). The BP5 bifurcation serves as organizing center for the system’s biological behavior, where two further curves of branching points emanate, labeled $\ell_{3}$ and $\ell_{4}$. The computed curves divides the parameter space into four zones as indicated in Fig 6(a). These regions characterize the asymptotic response of the HIV-COVID co-infection model (3) as described next. Region 1 (green): stable disease-free equilibrium; Region 2 (blue): COVID endemic equilibrium without HIV; Region 3 (yellow): HIV endemic equilibrium without COVID; Region 4 (red): full endemic equilibrium with both diseases. This behavior is numerically confirmed by the time plots computed at selected sample points in each region, see Fig 6(b).

## 5 Model extension with optimal control

Let us introduce four new control variables into our co–infection model in (3). The first control variable is the use of face masks to reduce the probability of COVID-19 infection. Individuals in *S*_*U*_ (the unaware compartment) are assumed to be unwilling to use face masks due to their lack of information about COVID-19. Let $u_{1}(t)\in [0,1]$ represent the proportion of individuals using face masks. Furthermore, let $\xi_{1}\in [0,1]$ denote the efficacy of face masks. The first group using face masks consists of susceptible aware individuals *S*_*A*_. Under this assumption, the infection rate of COVID-19 for *S*_*A*_ is given by:


$$(1-\xi_{1}u_{1}(t))f_{2}=\frac{\left(1-\xi_{1}u_{1}(t))\chi_{2}(C+\eta (C_{H}+C_{A})\right)}{N}.$$

The second group of individuals assumed to be using face masks consists of those in compartment *V*. Therefore, the infection term for *V* individuals is given by:


$$(1-\xi_{1}u_{1}(t))(1-\nu )f_{2}=\frac{\left(1-\xi_{1}u_{1}(t))(1-\nu )\chi_{2}(C+\eta (C_{H}+C_{A})\right)}{N}.$$

Individuals infected with HIV are assumed to be highly vulnerable to infections from other diseases. Hence, we assume that they are more aware of COVID-19 and voluntarily willing to use face masks. With this assumption, the infection term for individuals in compartment *H* with respect to COVID-19 is now expressed as:


$$(1-\xi_{1}u_{1})\Lambda_{1}f_{2}=\frac{\left(1-\xi_{1}u_{1})\Lambda_{1}\chi_{2}(C+\eta (C_{H}+C_{A})\right)}{N},$$

while for individuals in compartment *A*, it is given by:


$$(1-\xi_{1}u_{1})\Lambda_{2}f_{2}=\frac{\left(1-\xi_{1}u_{1})\Lambda_{2}\chi_{2}(C+\eta (C_{H}+C_{A})\right)}{N}.$$

The second control variable in our model is the vaccination rate, which was previously denoted by the constant β, and is now represented by *u*_2_(*t*). The third control variable in our model is the media campaign rate to increase public awareness of COVID-19, which was previously denoted by the constant θ, and is now represented by *u*_3_(*t*). The fourth control variable in our model, denoted by *u*_4_(*t*) (where $u_{4}(t)\in [0,1]$), represents the compliance rate to the usage of condom to prevent susceptible individuals from acquiring HIV infection. Furthermore, let $\xi_{2}\in [0,1]$ denote the efficacy of condom usage. Hence, the force of infection for HIV is given as


$$(1-\xi_{2}u_{4}(t))f_{1}=\frac{(1-\xi_{2}u_{4}(t))\chi_{1}\left(H+\omega (A+C_{H}+C_{A})\right)}{N}.$$

With this assumption, the optimal control model of co–infection between HIV and COVID-19 under these control measures is given by:

$$S_{U}^{\prime }=\Pi -((1-\xi_{2}u_{4}(t))f_{1}+\psi f_{2}+\mu +u_{3}(t))S_{U}+\varphi S_{A},\;S_{A}^{\prime }=u_{3}(t)S_{U}-((1-\xi_{2}u_{4}(t))f_{1}+(1-\xi_{1}u_{1}(t))f_{2}+\mu +\varphi +u_{2}(t))S_{A},\;V^{\prime }=u_{2}(t)S_{A}-((1-\xi_{2}u_{4}(t))f_{1}+(1-\xi_{1}u_{1}(t))(1-\nu )f_{2}+\mu )V,\;C^{\prime }=\psi f_{2}S_{U}+(1-\xi_{1}u_{1}(t))f_{2}S_{A}+(1-\xi_{1}u_{1}(t))(1-\nu )f_{2}V\ldots \;-((1-\xi_{2}u_{4}(t))f_{1}+\phi_{1}+\mu +\delta_{1})C,\;R^{\prime }=\phi_{1}C-((1-\xi_{2}u_{4}(t))f_{1}+\mu )R,\;H^{\prime }=(1-\xi_{2}u_{4}(t))f_{1}\left(S_{U}+S_{A}+V+R\right)+\phi_{2}C_{H}-(\mu +(1-\xi_{1}u_{1}(t))\Lambda_{1}f_{2}+\gamma_{1})H,\;A^{\prime }=\gamma_{1}H+\phi_{3}C_{A}-((1-\xi_{1}u_{1}(t))\Lambda_{2}f_{2}+\mu +\delta_{2})A,\;C_{H}^{\prime }=(1-\xi_{2}u_{4}(t))f_{1}C+(1-\xi_{1}u_{1}(t))\Lambda_{1}f_{2}H-(\phi_{2}+\gamma_{2}+\mu +\delta_{1})C_{H},\;C_{A}^{\prime }=\gamma_{2}C_{H}+(1-\xi_{1}u_{1}(t))\Lambda_{2}f_{2}A-(\phi_{3}+\mu +\delta_{3})C_{A}.$$ (24)

For this, we consider the following objective functional

$$𝒥(u_{1},u_{2},u_{3},u_{4})=\int_{0}^{t_{f}}=[b_{1}C+b_{2}H+b_{3}A+b_{4}C_{H}+b_{5}C_{A}\;+\frac{1}{2}\left(b_{6}u_{1}^{2}(t)+b_{7}u_{2}^{2}(t)+b_{8}u_{3}^{2}(t)+b_{9}u_{4}^{2}(t)\right)]dt.$$ (25)

Here, the final time is denoted by *t*_*f*_, and the weight constants help to balance each term in the integrand (25). The term $b_{1}C+b_{2}H+b_{3}A+b_{4}C_{H}+b_{5}C_{A}$, represents the cost associated with monitoring the infected individuals at all stages. The term $b_{6}u_{1}^{2}(t)+b_{7}u_{2}^{2}(t)+b_{8}u_{3}^{2}(t)$, denotes the cost associated with the public enlightenment campaign to educate the general public on the dynamics of COVID-19, implementing vaccination, and the cost of acquiring a face mask. The term $b_{9}u_{4}^{2}(t)$, represents the cost associated with the public health education campaign to educate the general public on safer sex practices.

The goal is to reduce the number of infected individuals as well as the cost associated with the control strategies. The aim is to find an optimal control quadruple *u*_1_(*t*), *u*_2_(*t*), *u*_3_(*t*) and *u*_4_(*t*) such that


$$𝒥[u_{1}^{*},u_{2}^{*},u_{3}^{*},u_{4}^{*}]=\min_{u_{1},u_{2},u_{3},u_{4}\in \Theta^{*}}𝒥[u_{1},u_{2},u_{3},u_{4}],$$

where the control set ($\Theta^{*}$) is defined as


$$\Theta^{*}=\{\left(u_{1}(t),u_{2}(t),u_{3}(t),u_{4}(t)\right)\in {ℒ}^{1}(0,t_{f})|a_{1}\leq u_{1}(t)\geq b_{1},a_{2}\leq u_{2}(t)\geq b_{2},a_{3}\leq u_{3}(t)\geq b_{3},\;a_{4}\leq u_{4}(t)\geq b_{4}\},$$

is Lesbgue measurable.

### 5.1 Analysis of the optimal control model

An optimal control pair must satisfy the necessary conditions specified by Pontryagin’s Maximum Principle (PMP) [57]. This principle convert (24) and (25) into a problem of minimizing pointwise a Hamiltonian (H) , with respect to the control quadruple *u*_1_(*t*), *u*_2_(*t*), *u*_3_(*t*) and *u*_4_(*t*). The Hamiltonian is given by;


$$H=b_{1}C+b_{2}H+b_{3}A+b_{4}C_{H}+b_{5}C_{A}+\frac{1}{2}\left(b_{6}u_{1}^{2}(t)+b_{7}u_{2}^{2}(t)+b_{8}u_{3}^{2}(t)+b_{9}u_{4}^{2}(t)\right)\;+\lambda_{1}\left[\Pi -((1-\xi_{2}u_{4}(t))f_{1}+\psi f_{2}+\mu +u_{3}(t))S_{U}+\varphi S_{A}\right]\;+\lambda_{2}\left[u_{3}(t)S_{U}-((1-\xi_{2}u_{4}(t))f_{1}+(1-\xi_{1}u_{1}(t))f_{2}+\mu +\varphi +u_{2}(t))S_{A}\right]\;+\lambda_{3}\left[u_{2}(t)S_{A}-((1-\xi_{2}u_{4}(t))f_{1}+(1-\xi_{1}u_{1}(t))(1-\nu )f_{2}+\mu )V\right]\;+\lambda_{4}[\psi f_{2}S_{U}+(1-\xi_{1}u_{1}(t))f_{2}S_{A}+(1-\xi_{1}u_{1}(t))(1-\nu )f_{2}V\ldots \;-((1-\xi_{2}u_{4}(t))f_{1}+\phi_{1}+\mu +\delta_{1})C]\;+\lambda_{5}\left[\phi_{1}C-((1-\xi_{2}u_{4}(t))f_{1}+\mu )R\right]\;+\lambda_{6}\left[(1-\xi_{2}u_{4}(t))f_{1}\left(S_{U}+S_{A}+V+R\right)+\phi_{2}C_{H}-(\mu +(1-\xi_{1}u_{1}(t))\Lambda_{1}f_{2}+\gamma_{1})H\right]\;+\lambda_{7}\left[\gamma_{1}H+\phi_{3}C_{A}-((1-\xi_{1}u_{1}(t))\Lambda_{2}f_{2}+\mu +\delta_{2})A\right]\;+\lambda_{8}\left[(1-\xi_{2}u_{4}(t))f_{1}C+(1-\xi_{1}u_{1}(t))\Lambda_{1}f_{2}H-(\phi_{2}+\gamma_{2}+\mu +\delta_{1})C_{H}\right]\;+\lambda_{9}\left[\gamma_{2}C_{H}+(1-\xi_{1}u_{1}(t))\Lambda_{2}f_{2}A-(\phi_{3}+\mu +\delta_{3})C_{A}\right],$$

where $\lambda_{1}$, $\lambda_{2}$, $\lambda_{3}$, $\lambda_{4}$, $\lambda_{5}$, $\lambda_{6}$, $\lambda_{7}$, $\lambda_{8}$ and $\lambda_{9}$ are the adjoint functions associated with the state variables of the model (24).

**Theorem 6.** *Given the optimal control sets $u_{1}^{*},u_{2}^{*},u_{3}^{*},u_{4}^{*}$ and the solutions $S_{U}^{*}$, $S_{A}^{*}$, ${\text{V}}^{*}$, ${\text{C}}^{*}$, ${\text{R}}^{*}$, ${\text{H}}^{*}$, ${\text{A}}^{*}$, $C_{H}^{*}$, $C_{A}^{*}$ of the corresponding state system (*24*) that minimizes $𝒥(u_{1},u_{2},u_{3},u_{4})$ over $\Theta^{*}$, then there exists adjoint functions $\lambda_{1}$, $\lambda_{2}$, $\lambda_{3}$, $\lambda_{4}$, $\lambda_{5}$, $\lambda_{6}$, $\lambda_{7}$, $\lambda_{8}$ and $\lambda_{9}$, such that*

$$\frac{d\lambda_{1}}{dt}=P_{1},\;\;\;\;\;\;\;\;\frac{d\lambda_{2}}{dt}=P_{2},\;\;\;\;\;\;\;\;\frac{d\lambda_{3}}{dt}=P_{3},\;\;\;\;\;\;\;\;\frac{d\lambda_{4}}{dt}=P_{4},\;\;\;\;\;\;\;\;\frac{d\lambda_{5}}{dt}=P_{5},\;\frac{d\lambda_{6}}{dt}=P_{6},\;\;\;\;\;\;\;\;\frac{d\lambda_{7}}{dt}=P_{7},\;\;\;\;\;\;\;\;\frac{d\lambda_{8}}{dt}=P_{8},\;\;\;\;\;\;\;\;\frac{d\lambda_{9}}{dt}=P_{9}.$$ (26)

*The expression for $P_{i}$ (for i=1,2,3,...,9) are given in S1 File. Furthermore, the transversality conditions is given by*

$$\lambda_{i}(t_{f})=0,\;i=1,2,3,...,9.$$ (27)

*The following characterization holds;*

$$u_{1}^{*}(t)=\max\left\{0,\min\left(1,\frac{1}{b_{6}}\left(L_{1}\right)\right)\right\},\;u_{2}^{*}(t)=\max\left\{0,\min\left(1,\frac{1}{b_{7}}(\lambda_{2}-\lambda_{3})S_{A}\right)\right\},\;u_{3}^{*}(t)=\max\left\{0,\min\left(1,\frac{1}{b_{8}}(\lambda_{1}-\lambda_{2})S_{U}\right)\right\},\;u_{4}^{*}(t)=\max\left\{0,\min\left(1,\frac{1}{b_{9}}\left(L_{2}\right)\right)\right\},$$ (28)

*where*


$$L_{1}=\frac{\xi_{1}\chi_{2}\left(C+\eta (C_{H}+C_{A})\right)\Lambda_{1}}{N}(\Lambda_{2}A(\lambda_{9}-\lambda_{7})+\Lambda_{1}H(\lambda_{8}-\lambda_{6})\;+\lambda_{4}(S_{A}+(1-\nu )V)-\lambda_{3}(1-\nu )V-\lambda_{2}S_{A}),\;L_{2}=\frac{\xi_{2}\chi_{1}\left(H+\omega (A+C_{H}+C_{A})\right)}{N}((\lambda_{8}-\lambda_{4})C+\lambda_{6}(S_{U}+S_{A}+V+R)\;-\lambda_{5}R-\lambda_{3}V-\lambda_{2}S_{A}-\lambda_{1}S_{U}).$$

**Preposition 1** *Corollary 4.2 of (Fleming and Rishel [58] gives the existence of an optimal control sets (*u**_*1*_*(*t*), *u**_*2*_*(*t*), *u**_*3*_*(*t*) and *u**_*4*_*(*t*)) due to the convexity of the integrand of J with respect of (*u**_*1*_*(*t*), *u**_*2*_*(*t*), *u**_*3*_*(*t*) and *u**_*4*_*(*t*)), a prior boundedness of the state solutions, and the local Lipschitz property of the model (*24*) with respect to the variables.*

*Proof:* Using the Pontryagin’s Maximum Principles, we obtained


$$\frac{d\lambda_{1}}{dt}=-\frac{\partial H}{\partial S_{U}},\;\lambda_{1}(t_{f})=0,\;\;\;\;\;\;\;\;\frac{d\lambda_{2}}{dt}=-\frac{\partial H}{\partial S_{A}},\;\lambda_{2}(t_{f})=0,\;\;\;\;\;\;\;\;\frac{d\lambda_{3}}{dt}=-\frac{\partial H}{\partial V},\;\lambda_{3}(t_{f})=0,\;\frac{d\lambda_{4}}{dt}=-\frac{\partial H}{\partial C},\;\lambda_{4}(t_{f})=0,\;\;\;\;\;\;\;\;\frac{d\lambda_{5}}{dt}=-\frac{\partial H}{\partial R},\;\lambda_{5}(t_{f})=0,\;\;\;\;\;\;\;\;\frac{d\lambda_{6}}{dt}=-\frac{\partial H}{\partial H},\;\lambda_{6}(t_{f})=0,\;\frac{d\lambda_{7}}{dt}=-\frac{\partial H}{\partial A},\;\lambda_{7}(t_{f})=0,\;\;\;\;\;\;\;\;\frac{d\lambda_{8}}{dt}=-\frac{\partial H}{\partial C_{H}},\;\lambda_{8}(t_{f})=0,\;\;\;\;\;\;\;\;\frac{d\lambda_{9}}{dt}=-\frac{\partial H}{\partial C_{A}},\;\lambda_{9}(t_{f})=0,\;$$

and considering the optimality condition;


$$\frac{\partial H}{\partial u_{1}}=0,\frac{\partial H}{\partial u_{2}}=0,\frac{\partial H}{\partial u_{3}}=0,\;\;\;\text{and}\;\;\;\;\frac{\partial H}{\partial u_{4}}=0.$$

This optimal control sets(*u*_1_(*t*), *u*_2_(*t*), *u*_3_(*t*) and *u*_4_(*t*)) can be solved for subject to the state variables. Taking into account the bounds on the controls, the characterization can be solved as follows;

For the control *u*_1_(*t*), we have


$$\frac{\partial H}{\partial u_{1}}=u_{1}b_{6}-\frac{\xi_{1}\chi_{2}\left(C+\eta (C_{H}+C_{A})\right)\Lambda_{1}}{N}(\Lambda_{2}A(\lambda_{9}-\lambda_{7})+\Lambda_{1}H(\lambda_{8}-\lambda_{6})\;+\lambda_{4}(S_{A}+(1-\nu )V)-\lambda_{3}(1-\nu )V-\lambda_{2}S_{A})=0,$$

so that


$$u_{1}^{*}(t)=\frac{\xi_{1}\chi_{2}\left(C+\eta (C_{H}+C_{A})\right)\Lambda_{1}}{b_{6}N}(\Lambda_{2}A(\lambda_{9}-\lambda_{7})+\Lambda_{1}H(\lambda_{8}-\lambda_{6})\;+\lambda_{4}(S_{A}+(1-\nu )V)-\lambda_{3}(1-\nu )V-\lambda_{2}S_{A}).$$

For the control *u*_2_(*t*), we have


$$\frac{\partial H}{\partial u_{2}}=u_{2}b_{7}-S_{A}\lambda_{2}+S_{A}\lambda_{3}=0.$$

Solve it respect to *u*_2_ gives


$$u_{2}^{*}(t)=\frac{1}{b_{7}}\left(\lambda_{2}-\lambda_{3}\right)S_{A}.$$

Next, taking the derivative of H respect to *u*_3_ gives


$$\frac{\partial H}{\partial u_{3}}=u_{3}b_{8}-S_{U}\lambda_{1}+S_{U}\lambda_{2}=0.$$

Similarly, solve it respect to *u*_3_ yield


$$u_{3}^{*}(t)=\frac{1}{b_{8}}\left(\lambda_{1}-\lambda_{2}\right)S_{U}.$$

For the control *u*_4_(*t*), we have


$$\frac{\partial H}{\partial u_{4}}=u_{4}b_{9}-\frac{\xi_{2}\chi_{1}\left(H+\omega (A+C_{H}+C_{A})\right)}{N}((\lambda_{8}-\lambda_{4})C+\lambda_{6}(S_{U}+S_{A}+V+R)\;-\lambda_{5}R-\lambda_{3}V-\lambda_{2}S_{A}-\lambda_{1}S_{U})=0.$$

Thus, we obtained


$$u_{4}^{*}(t)=\frac{\xi_{2}\chi_{1}\left(H+\omega (A+C_{H}+C_{A})\right)}{b_{9}N}((\lambda_{8}-\lambda_{4})C+\lambda_{6}(S_{U}+S_{A}+V+R)\;-\lambda_{5}R-\lambda_{3}V-\lambda_{2}S_{A}-\lambda_{1}S_{U}).$$

Clearly, the optimality conditions obtained by taking the derivatives of the Hamiltonian with respect to the controls on hold in the interior of the control set. This end the proof. ◻

### 5.2 Numerical illustration of the control model

The forward-backward sweep approach is often utilized to determine the optimal control solution. The method begins with an initial estimate of the control variables. Using this initial guess, the state equations are integrated forward in time with a fourth-order Runge-Kutta technique. After the forward integration is complete, the resulting state trajectories, coupled with the initial control estimate, are used to solve the adjoint equations. These adjoint equations are integrated backward in time, starting with the stated terminal conditions. The iterative technique is performed until convergence is reached. This backward time integration is carried out using the reverse fourth-order Runge-Kutta method. The control variables, denoted as *u*_1_(*t*), *u*_2_(*t*), *u*_3_(*t*), and *u*_4_(*t*), are then updated and used to resolve both the state and adjoint systems again. This iterative process continues until satisfactory convergence is achieved in the state, adjoint, and control variables, as described in previous studies [59–62]. The parameter values used in the numerical illustration are specifically those outlined in Table 1. The initial conditions used for the state variables are *S*_*U*_(0) = 9900, *S*_*A*_(0) = 0, V(0)=0, C(0)=50, R(0)=0, H(0)=30, A(0)=20, *C*_*H*_(0) = 0, and *C*_*A*_(0) = 0. The values of the weight factors used are *b*_1_ = 1, *b*_2_ = 1, *b*_3_ = 1, *b*_4_ = 1, *b*_5_ = 1, *b*_6_ = 1, *b*_7_ = 1, *b*_8_ = 1, and *b*_9_ = 1. The control strategies are combination of efforts involving the following

1. Strategy A: combination of *u*_1_(*t*), *u*_3_(*t*), and *u*_4_(*t*), while setting *u*_2_ to zero.
2. Strategy B: combination of *u*_2_(*t*), *u*_3_(*t*), and *u*_4_(*t*), while setting *u*_1_ to zero.
3. Strategy C: combination of *u*_1_(*t*), *u*_2_(*t*), *u*_3_(*t*), and *u*_4_(*t*).

**Fig 7:**
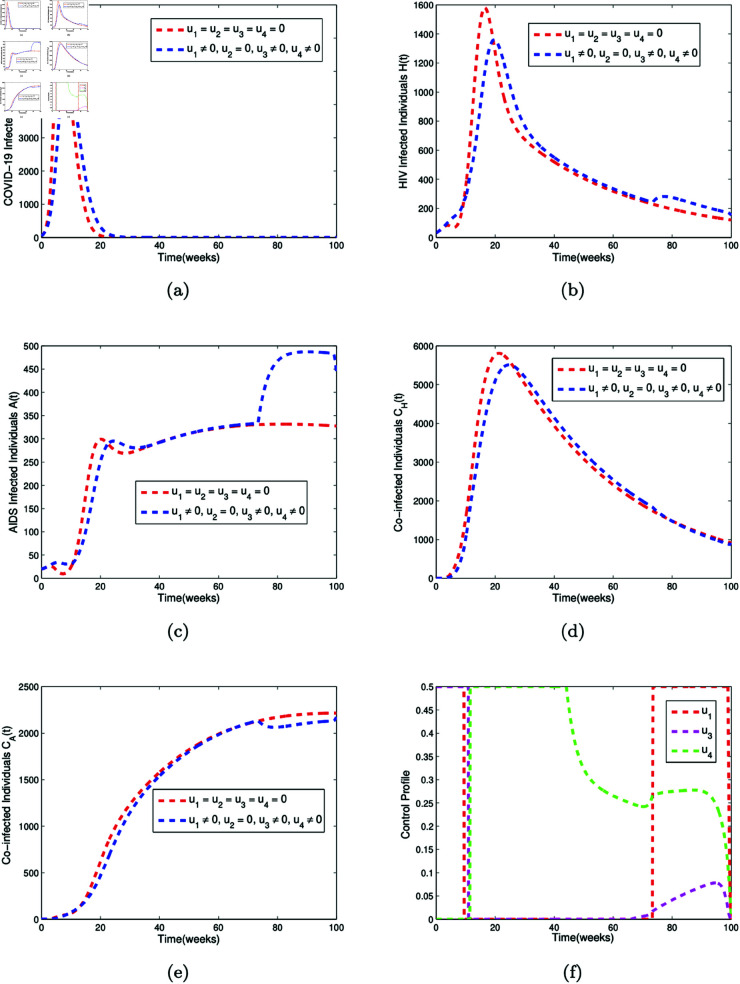
Optimal control simulation for Strategy A. Simulation of (a) the effect of the control profiles *u*_1_(*t*), *u*_3_(*t*), and *u*_4_(*t*) on COVID-19 infected individuals (b) the effect of the control profiles *u*_1_(*t*), *u*_3_(*t*), and *u*_4_(*t*) on HIV infected individuals (c) the effect of the control profiles *u*_1_(*t*), *u*_3_(*t*), and *u*_4_(*t*) on AIDS infected individuals (d) the effect of the control profiles *u*_1_(*t*), *u*_3_(*t*), and *u*_4_(*t*) on individuals co-infected with both COVID-19 and HIV (e) the effect of the control profiles *u*_1_(*t*), *u*_3_(*t*), and *u*_4_(*t*) on individuals co-infected with both COVID-19 and AIDS (f) the control profiles *u*_1_(*t*), *u*_3_(*t*), and *u*_4_(*t*).

**Fig 8:**
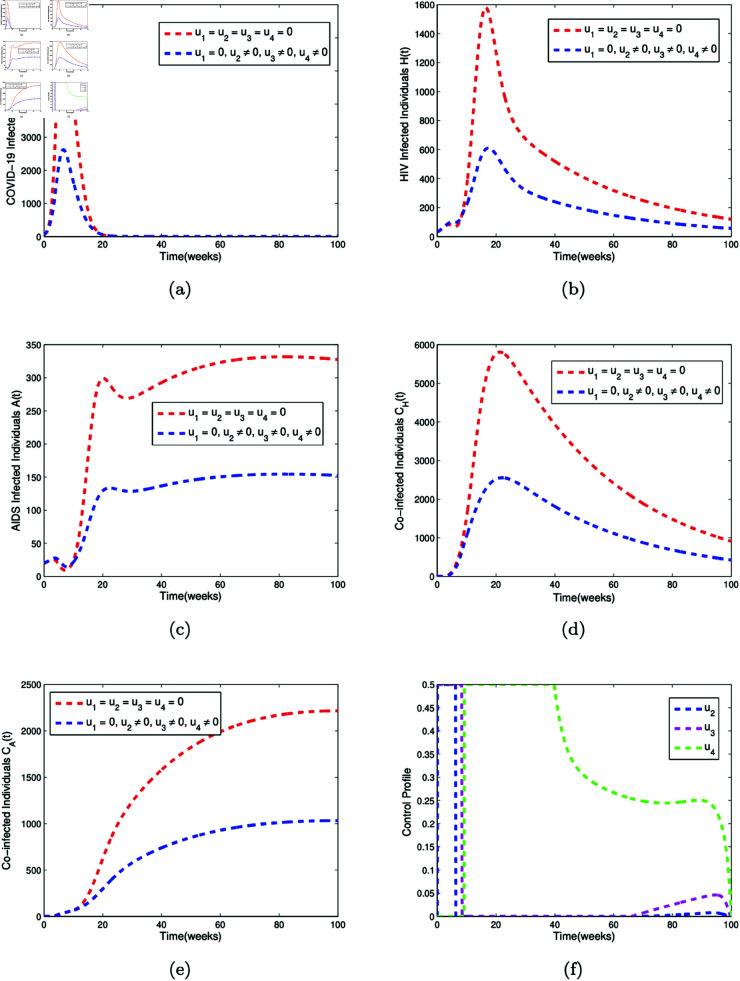
Optimal control simulation for Strategy B. Simulation of (a) the effect of the control profiles *u*_2_(*t*), *u*_3_(*t*), and *u*_4_(*t*) on COVID-19 infected individuals (b) the effect of the control profiles *u*_2_(*t*), *u*_3_(*t*), and *u*_4_(*t*) on HIV infected individuals (c) the effect of the control profiles *u*_2_(*t*), *u*_3_(*t*), and *u*_4_(*t*) on AIDS infected individuals (d) the effect of the control profiles *u*_2_(*t*), *u*_3_(*t*), and *u*_4_(*t*) on individuals co-infected with both COVID-19 and HIV (e) the effect of the control profiles *u*_2_(*t*), *u*_3_(*t*), and *u*_4_(*t*) on individuals co-infected with both COVID-19 and AIDS (f) the control profiles *u*_2_(*t*), *u*_3_(*t*), and *u*_4_(*t*).

**Fig 9:**
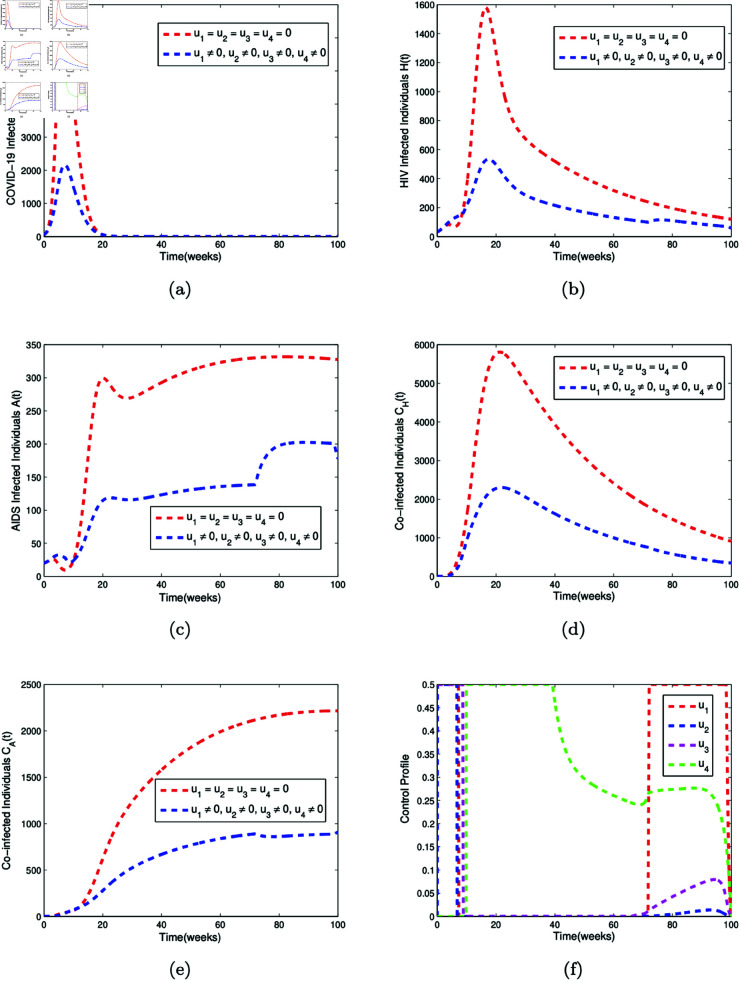
Optimal control simulation for Strategy C. Simulation of (a) the effect of the control profiles *u*_1_(*t*), *u*_2_(*t*), *u*_3_(*t*), and *u*_4_(*t*) on COVID-19 infected individuals (b) the effect of the control profiles *u*_1_(*t*), *u*_2_(*t*), *u*_3_(*t*), and *u*_4_(*t*) on HIV infected individuals (c) the effect of the control profiles *u*_1_(*t*), *u*_2_(*t*), *u*_3_(*t*), and *u*_4_(*t*) on AIDS infected individuals (d) the effect of the control profiles *u*_1_(*t*), *u*_2_(*t*), *u*_3_(*t*), and *u*_4_(*t*) on individuals co-infected with both COVID-19 and HIV (e) the effect of the control profiles *u*_1_(*t*), *u*_2_(*t*), *u*_3_(*t*), and *u*_4_(*t*) on individuals co-infected with both COVID-19 and AIDS (f) the control profiles *u*_1_(*t*), *u*_2_(*t*), *u*_3_(*t*), and *u*_4_(*t*).

The numerical simulations for Strategy A, B, and C are given in Figs 7, 8, and 9, respectively.

### 5.3 Discussion of results

Fig 7 is the simulation of the effect of control strategy A, which comprises of the usage of face mask (*u*_1_(*t*)), media campaign rate (*u*_3_(*t*)), and the compliance rate to condom usage (*u*_4_(*t*)) on the COVID-19 infected compartment (*C*(*t*)), HIV infected compartment (*H*(*t*)), AIDS infected compartment (*A*(*t*)), co-infected compartment with COVID-19 and HIV (*C*_*H*_(*t*)), and co-infected compartment with COVID-19 and AIDS (*C*_*A*_(*t*)). When control strategy A is applied, the peak number of COVID-19 infected individuals decreases from 6800 to 5300 by week 20, as shown in Fig 7(a). For HIV infected individuals, the peak value drops from 1600 to 1350 by week 30, then rises from 200 to 300 by week 80, as depicted in Fig 7(b). Fig 7(c) shows that the number of individuals infected with AIDS increases from approximately 300 to 470 by week 80 under strategy A. Fig 7(d) illustrates that the peak number of individuals co-infected with both COVID-19 and HIV decreases from about 5800 to 5400 by week 30. Additionally, Fig 7(e) demonstrates that the number of individuals co-infected with both COVID-19 and AIDS reduces from around 2200 to 1800 by week 80 when control strategy A is implemented. Fig 7(f) is the corresponding controls *u*_1_(*t*), *u*_3_(*t*), and *u*_4_(*t*)

Fig 8 presents the simulation of the effect of control strategy B, which comprises of the COVID-19 vaccination rate (*u*_2_(*t*)), media campaign rate (*u*_3_(*t*)), and the compliance rate to condom usage (*u*_4_(*t*)) on the COVID-19 infected compartment (*C*(*t*)), HIV infected compartment (*H*(*t*)), AIDS infected compartment (*A*(*t*)), co-infected compartment with COVID-19 and HIV (*C*_*H*_(*t*)), and co-infected compartment with COVID-19 and AIDS (*C*_*A*_(*t*)). When control strategy B is implemented, it is observed that the peak number of COVID-19 infected individuals drops from 6800 to 2700 by week 20, as shown in Fig 8(a). The peak number of HIV infected individuals decreases from 1600 to 610 by week 20, as depicted in Fig 8(b). In Fig 8(c), it is seen that the number of AIDS infected individuals falls from 330 to below 150 between week 20 and week 100. Fig 8(d) shows that the peak number of co-infected individuals with both COVID-19 and HIV decreases from around 5800 to 2600 by week 30 under strategy B. Additionally, Fig 8(e) illustrates that the number of co-infected individuals with both COVID-19 and AIDS drops from approximately 2200 to 900 by week 80 when control strategy B is implemented. Fig 8(f) is the corresponding controls *u*_2_(*t*), *u*_3_(*t*), and *u*_4_(*t*).

Fig 9 is the simulation of the effect of control strategy C, which comprises of the usage of face mask (*u*_1_(*t*)), the COVID-19 vaccination rate (*u*_2_(*t*)), media campaign rate (*u*_3_(*t*)), and the compliance rate to condom usage (*u*_4_(*t*)) on the COVID-19 infected compartment (*C*(*t*)), HIV infected compartment (*H*(*t*)), AIDS infected compartment (*A*(*t*)), co-infected compartment with COVID-19 and HIV (*C*_*H*_(*t*)), and co-infected compartment with COVID-19 and AIDS (*C*_*A*_(*t*)). When control strategy C is implemented, it is observed that the peak number of COVID-19 infected individuals decreases from 6800 to 2100 by week 20, as shown in Fig 9(a). The peak number of HIV infected individuals reduces from approximately 1600 to 505 by week 20, as depicted in Fig 9(b). In Fig 9(c), it is observed that the number of AIDS infected individuals decreases from 330 to 200 by week 100 under strategy C. Fig 9(d) shows that the peak number of co-infected individuals with both COVID-19 and HIV drops from around 5800 to 2300 by week 30 when strategy C is applied. Additionally, Fig 9(e) illustrates that the number of co-infected individuals with both COVID-19 and AIDS decreases from around 2200 to 750 by week 80 under control strategy C. Fig 9(f) is the corresponding controls *u*_1_(*t*), *u*_2_(*t*), *u*_3_(*t*), and *u*_4_(*t*).

Our results indicate that the most effective strategy to mitigate and control the spread of COVID-19, HIV/AIDS, and their co-infection is the combination of the four control measures (Strategy C). This strategy includes the use of face masks, COVID-19 vaccination, media campaigns to raise public awareness about COVID-19, and compliance with condom usage.

Our optimal control simulations demonstrate that integrated intervention strategies can significantly reduce the morbidity associated with HIV and COVID-19 co-infections. For COVID-19, the combined implementation of face masks, vaccination, and public awareness campaigns substantially decreases transmission, lowers the peak number of infections, and shortens the duration of the epidemic. For HIV, condom use effectively reduces new infections over time. The simulations reveal that simultaneous implementation of all available interventions leads to the greatest impact—minimizing both the individual and combined disease burdens. These results emphasize that partial or isolated interventions are insufficient in controlling co-infections. Instead, long-term, coordinated public health strategies are required to achieve meaningful reductions in disease morbidity. Our findings provide quantitative support for prioritizing comprehensive intervention packages in policy planning, especially in regions affected by syndemic conditions.

## 6 Conclusion and future works

Mathematical models have been extensively utilized to understand the mechanisms of various disease transmissions within populations, including HIV, COVID-19, and their co-infection. In modeling HIV transmission, numerous studies have examined the impact of antiretroviral therapy [63–65], age-structured populations [21, 22, 66], community awareness [23, 67], co-infections with other diseases [37, 68, 69], and various other factors. For COVID-19, since its emergence as a world-scale pandemic in 2020, a substantial body of scientific articles has focused on mathematical models of its transmission. Topics of interest include vaccination strategies, the effects of population awareness, variations in infection rates, the dynamics of multiple viral strains, and more [70–75]. Co-infection models have also been explored by many authors, particularly regarding HIV or COVID-19 with other diseases. However, only a few studies have specifically addressed mathematical models for co-infection between HIV and COVID-19 [46, 46–48, 48, 50]. According to the explanation above, there are several reasons why studying HIV and COVID-19 co-infection using mathematical models is essential. First, the immune responses of individuals living with HIV may influence the course, spread, and outcomes of COVID-19. A mathematical model provides a framework for quantitatively investigating these interactions in order to gain a better understanding of the dynamics of co-infection. Additionally, mathematical models could evaluate the combined impacts of treatments such as the COVID-19 vaccine, offering insights on the most effective strategies to treat both illnesses simultaneously.

As mentioned before, mathematical models on the co–infection between HIV and COVID-19 are not so many discussed by mathematician. Furthermore, during post pandemic of COVID-19, the importance of community awareness becomes more essential since public awareness on COVID-19 are not so much high as during pandemic era. Hence, it is essential to discuss the impact of public awareness on the contagiousness of COVID-19 considering co–infections with HIV.

Hence, in this study we formulated and analyzed a deterministic epidemic co–infection model between HIV and COVID-19 in a population. The model was constructed as a system of ordinary differential equations. Our discussion focuses on a qualitative investigation of the HIV only model, COVID-19 only model and the co–infection model. Existence and local stability criteria of various types of equilibrium points appearing in the model were derived based on the bifurcation theorem in [53]. The main findings of this study are given as follows.

1. *The model with HIV only*. Our work shows that the HIV-free steady state is locally asymptotically stable if the basic reproduction number for HIV, denoted by ${ℛ}_{0_{HIV}}$ (6), is less than one. Furthermore, ${ℛ}_{0_{HIV}}=1$ serves as the threshold, where the stability of the HIV-free steady state changes from stable to unstable when ${ℛ}_{0_{HIV}}>1$, and, at the same time, the HIV-endemic equilibrium begins to emerge. This result emphasizes the importance of keeping ${ℛ}_{0_{HIV}}$ below one through effective interventions to reduce the infection rate such as widespread of HIV awareness program and behavioral prevention strategies. Once ${ℛ}_{0_{HIV}}$ exceeds one, the infection becomes self-sustaining in the population, indicating a potential for long-term persistence and increased disease burden. Therefore, identifying and targeting the factors that influence ${ℛ}_{0_{HIV}}$ is critical for controlling and eventually eliminating HIV transmission.
2. *The model with COVID-19 only*. Our dynamical analysis shows that the COVID-19-free steady state is locally stable whenever the basic reproduction number for COVID-19, denoted by ${ℛ}_{0_{COV}}$ (12), is smaller than one, and unstable if it is bigger than one. The COVID-19 endemic steady state always exists, is unique, and is stable if ${ℛ}_{0_{COV}}>1$. Furthermore, a backward bifurcation at ${ℛ}_{0_{COV}}=1$ is possible due to the mortality rate induced by COVID-19. Consequently, a higher death rate induced by COVID-19 increases the likelihood of a stable COVID-19 endemic equilibrium even when ${ℛ}_{0_{COV}}<1$. This implies that simply reducing ${ℛ}_{0_{COV}}$ below one may not be sufficient to eradicate the disease if mortality-induced backward bifurcation occurs. Therefore, public health strategies must focus not only on reducing transmission but also on minimizing COVID-19-related mortality through timely treatment, improved health care access, and vaccination. Failure to address this could result in persistent endemicity despite reproduction numbers being nominally below unity.
3. *The co–infection model*. The basic reproduction number of the co-infection system is given by the maximum value between ${ℛ}_{0_{HIV}}$ and ${ℛ}_{0_{COV}}$ (23). Our numerical experiments using the software COCO reveal that the disease-free equilibrium (free of HIV and COVID-19) is locally stable if both ${ℛ}_{0_{HIV}}$ and ${ℛ}_{0_{COV}}$ are smaller than one. The single-disease endemic equilibrium may be stable only if ${ℛ}_{0_{HIV}}$ and ${ℛ}_{0_{COV}}$ exceeds one. Furthermore, the condition where ${ℛ}_{0_{HIV}}$ and ${ℛ}_{0_{COV}}$ both equal one serves as the organizing center for the dynamical behavior. Small changes around this condition can lead to significantly different dynamics in the co-infection model. This implies that even marginal increases in either reproduction number could trigger the persistence of one or both diseases in the population. As a result, public health strategies must be integrated and responsive to the co-infection context, as control measures for one disease may not be sufficient if the other remains above threshold. Coordinated intervention is essential to avoid overlapping epidemics and prevent a potential syndemic scenario.
4. *The optimal control model.* The study underscores the significance of effective control measures for HIV and COVID-19 co-infections. Face masks, vaccination, public awareness, and condom use are all effective approaches for reducing infection rates. Combined actions have a greater impact than isolated efforts, with vaccination and mask use reducing COVID-19 spread and condom use limiting HIV transmission. Public awareness enhances the effectiveness of interventions, emphasizing the importance of coordinating initiatives. Numerical results show that long-term, well-planned efforts can result in infection elimination or stabilization of infections. These findings suggest that integrated, multi-faceted public health campaigns are essential to effectively manage co-epidemics. Neglecting one aspect of control—such as awareness or preventive behavior—could undermine the success of even well-funded medical interventions.

Although our model serves an important insight to the co–infection phenomena between HIV and COVID-19 under some important intervention scenarios, we believe that our model still can be extended on several different direction. First direction is by exploring the model by considering varying levels of awareness among different sub-populations, such as adults versus children or urban versus rural communities. The next possible direction is to investigate a non-standard infection term to account for possible saturation effects in infection rates to accommodate people response on the disease transmission in a community. Incorporating stochastic elements into the model to capture random fluctuations in disease transmission dynamics also can be another option for future research. Last, we believe that utilizing more comprehensive datasets, particularly for HIV, to enhance parameter estimation and model validation are necessary for future research direction.

## Supporting information

S1 File The expression of the co-state system for the optimal control model. (PDF)
