# Supplementary material for: Reassessment of public awareness and prevention strategies for HIV and COVID-19 co-infections through epidemic modeling
Source: PLoS One. 2025 Jul 31;20(7):e0328488. doi: 10.1371/journal.pone.0328488 (PMC12312958; doi:10.1371/journal.pone.0328488)
Supplement: S1 File — (PDF) [file pone.0328488.s001.pdf]

S1 File. The expression of the co-state system for the optimal control model.

$$
\begin{aligned}
\mathscr{P}_1 = & -\lambda_1 \Bigg(\left((1-\xi_2 u_4(t))\Upsilon_1 + \psi\Upsilon_2 \right) S_U - \frac{(1-\xi_2 u_4(t))\chi_1 \left(H + \omega(A + C_H + C_A)\right)}{N} \\
& - \frac{\psi\chi_2 \left(C + \eta(C_H + C_A)\right)}{N} - \mu - u_3(t) \Bigg) - \lambda_2 \left(u_3(t) + \left((1-\xi_2 u_4(t))\Upsilon_1 + \right.\right. \\
& \left.\left. (1-\xi_1 u_1(t))\Upsilon_2 \right) S_A \right) - \lambda_3 \left((1-\xi_2 u_4(t))\Upsilon_1 + (1-\nu)(1-\xi_1 u_1(t))\Upsilon_2 \right) V - \\
& \lambda_4 \Bigg(\frac{\psi\chi_2 \left(C + \eta(C_H + C_A)\right)}{N} - \psi\Upsilon_2 S_U + (1-\xi_2 u_4(t))\Upsilon_1 C - \\
& (S_A + (1-\nu)V)(1-\xi_1 u_1(t))\Upsilon_2 \Bigg) - \lambda_5 (1-\xi_2 u_4(t))\Upsilon_1 R - \\
& \lambda_6 \Bigg(\frac{(1-\xi_2 u_4(t))\chi_1 \left(H + \omega(A + C_H + C_A)\right)}{N} + (1-\xi_1 u_1(t))\Upsilon_2 \Lambda_1 H - \\
& (1-\xi_2 u_4(t))\Upsilon_1 (S_U + S_A + V + R) \Bigg) + (1-\xi_1 u_1(t))\Upsilon_2 \Lambda_1 A (\lambda_9 - \lambda_7) + \\
& \lambda_8 \left((1-\xi_2 u_4(t))\Upsilon_1 C + (1-\xi_1 u_1(t))\Upsilon_2 \Lambda_1 H \right),
\end{aligned}
\tag{1}
$$

$$
\begin{aligned}
\mathscr{P}_2 = & -\lambda_1 \left(\left((1-\xi_2 u_4(t))\Upsilon_1 + \psi\Upsilon_2 \right) S_U + \varphi \right) - \lambda_2 \Bigg(- \frac{(1-\xi_1 u_1(t))\chi_2 \left(C + \eta(C_H + C_A)\right)}{N} \\
& + \left((1-\xi_2 u_4(t))\Upsilon_1 + (1-\xi_1 u_1(t))\Upsilon_2 \right) S_A - (\mu + \varphi + u_2(t)) - \\
& \frac{(1-\xi_2 u_4(t))\chi_1 \left(H + \omega(A + C_H + C_A)\right)}{N} \Bigg) - \lambda_3 \left(u_2(t) + \right. \\
& \left. \left((1-\xi_2 u_4(t))\Upsilon_1 + (1-\nu)(1-\xi_1 u_1(t))\Upsilon_2 \right) V \right) - \\
& \lambda_4 \Bigg(\frac{(1-\xi_1 u_1(t))\chi_2 \left(C + \eta(C_H + C_A)\right)}{N} - \psi\Upsilon_2 S_U + (1-\xi_2 u_4(t))\Upsilon_1 C - \\
& (S_A + (1-\nu)V)(1-\xi_1 u_1(t))\Upsilon_2 \Bigg) - \lambda_5 (1-\xi_2 u_4(t))\Upsilon_1 R - \\
& \lambda_6 \Bigg(\frac{(1-\xi_2 u_4(t))\chi_1 \left(H + \omega(A + C_H + C_A)\right)}{N} + (1-\xi_1 u_1(t))\Upsilon_2 \Lambda_1 H - \\
& (1-\xi_2 u_4(t))\Upsilon_1 (S_U + S_A + V + R) \Bigg) + (1-\xi_1 u_1(t))\Upsilon_2 \Lambda_1 A (\lambda_9 - \lambda_7) + \\
& \lambda_8 \left((1-\xi_2 u_4(t))\Upsilon_1 C + (1-\xi_1 u_1(t))\Upsilon_2 \Lambda_1 H \right),
\end{aligned}
\tag{2}
$$

$$
\begin{aligned}
\mathscr{P}_3 = & -\lambda_1 \left((1-\xi_2 u_4(t))\Upsilon_1 + \psi\Upsilon_2 \right) S_U - \lambda_2 \left((1-\xi_2 u_4(t))\Upsilon_1 + (1-\xi_1 u_1(t))\Upsilon_2 \right) S_A \\
& - \lambda_3 \Bigg(\left((1-\xi_2 u_4(t))\Upsilon_1 + (1-\nu)(1-\xi_1 u_1(t))\Upsilon_2 \right) V - \mu - \\
& \frac{(1-\nu)(1-\xi_1 u_1(t))\chi_2 \left(C + \eta(C_H + C_A)\right)}{N} - \\
& \frac{(1-\xi_2 u_4(t))\chi_1 \left(H + \omega(A + C_H + C_A)\right)}{N} \Bigg) - \\
& \lambda_4 \Bigg(\frac{(1-\nu)(1-\xi_1 u_1(t))\chi_2 \left(C + \eta(C_H + C_A)\right)}{N} - \psi\Upsilon_2 S_U + \\
& (1-\xi_2 u_4(t))\Upsilon_1 C - (S_A + (1-\nu)V)(1-\xi_1 u_1(t))\Upsilon_2 \Bigg) - \\
& \lambda_5 (1-\xi_2 u_4(t))\Upsilon_1 R - \lambda_6 \Bigg(\frac{(1-\xi_2 u_4(t))\chi_1 \left(H + \omega(A + C_H + C_A)\right)}{N} + \\
& (1-\xi_1 u_1(t))\Upsilon_2 \Lambda_1 H - (1-\xi_2 u_4(t))\Upsilon_1 (S_U + S_A + V + R) \Bigg) + \\
& (1-\xi_1 u_1(t))\Upsilon_2 \Lambda_1 A (\lambda_9 - \lambda_7) + \\
& \lambda_8 \left((1-\xi_2 u_4(t))\Upsilon_1 C + (1-\xi_1 u_1(t))\Upsilon_2 \Lambda_1 H \right),
\end{aligned}
\tag{3}
$$

$$
\begin{aligned}
\mathscr{P}_4 = &-b_1 - \lambda_1 \left(\frac{\psi\chi_2}{N} - (1-\xi_2 u_4(t))\Upsilon_1 - \psi\Upsilon_2 \right) S_U + \\
&\lambda_2 \left(\frac{(1-\xi_1 u_1(t))\chi_2}{N} - (1-\xi_2 u_4(t))\Upsilon_1 - (1-\xi_1 u_1(t))\Upsilon_2 \right) S_A + \\
&\lambda_3 \left(\frac{(1-\nu)(1-\xi_1 u_1(t))\chi_2}{N} - (1-\xi_2 u_4(t))\Upsilon_1 - (1-\nu)(1-\xi_1 u_1(t))\Upsilon_2 \right) V \\
&- \lambda_4 \left(\frac{\psi\chi_2 S_U}{N} - \psi\Upsilon_2 S_U - (S_A + (1-\nu)V)(1-\xi_1 u_1(t))\Upsilon_2 + \right. \\
&\frac{(S_A + (1-\nu)V)(1-\xi_1 u_1(t))\chi_2}{N} + (1-\xi_2 u_4(t))\Upsilon_1 C - \\
&\left. \frac{(1-\xi_2 u_4(t))\chi_1 \left(H + \omega(A + C_H + C_A)\right)}{N} - (\phi_1 + \mu + \delta_1) \right) - \\
&\lambda_5 \left(\phi_1 + (1-\xi_2 u_4(t))\Upsilon_1 R\right) + \lambda_6 \left(\left(\frac{(1-\xi_1 u_1(t))\chi_2 \Lambda_1}{N} - \right.\right. \\
&\left. (1-\xi_1 u_1(t))\Upsilon_2 \Lambda_1 \right) H + (1-\xi_2 u_4(t))\Upsilon_1 (S_U + S_A + V + R) \Big) + \\
&(\lambda_7 - \lambda_9) \left(\frac{(1-\xi_1 u_1(t))\chi_2 \Lambda_2}{N} - (1-\xi_1 u_1(t))\Upsilon_2 \Lambda_2 \right) A + \\
&\lambda_8 \Big((1-\xi_2 u_4(t))\Upsilon_1 C + (1-\xi_1 u_1(t))\Upsilon_2 \Lambda_1 H - \\
&\left. \frac{(1-\xi_2 u_4(t))\chi_1 \left(H + \omega(A + C_H + C_A)\right)}{N} - \frac{(1-\xi_1 u_1(t))\chi_2 \Lambda_1 H}{N} \right),
\end{aligned} \tag{4}
$$

$$
\begin{aligned}
\mathscr{P}_5 = &-\lambda_1 \left((1-\xi_2 u_4(t))\Upsilon_1 + \psi\Upsilon_2\right) S_U - \lambda_2 \left((1-\xi_2 u_4(t))\Upsilon_1 + (1-\xi_1 u_1(t))\Upsilon_2\right) S_A \\
&- \lambda_3 \left((1-\xi_2 u_4(t))\Upsilon_1 + (1-\nu)(1-\xi_1 u_1(t))\Upsilon_2\right) V - \\
&\lambda_4 \left((1-\xi_2 u_4(t))\Upsilon_1 C - \psi\Upsilon_2 S_U - (S_A + (1-\nu)V)(1-\xi_1 u_1(t))\Upsilon_2\right) - \\
&\lambda_5 \left((1-\xi_2 u_4(t))\Upsilon_1 R - \frac{(1-\xi_2 u_4(t))\chi_1 \left(H + \omega(A + C_H + C_A)\right)}{N} - \mu \right) - \\
&\lambda_6 \left(\frac{(1-\xi_2 u_4(t))\chi_1 \left(H + \omega(A + C_H + C_A)\right)}{N} + (1-\xi_1 u_1(t))\Upsilon_2 \Lambda_1 - \right. \\
&(1-\xi_2 u_4(t))\Upsilon_1 (S_U + S_A + V + R) \Big) + (1-\xi_1 u_1(t))\Upsilon_2 \Lambda_1 A (\lambda_9 - \lambda_7) + \\
&\lambda_8 \left((1-\xi_2 u_4(t))\Upsilon_1 C + (1-\xi_1 u_1(t))\Upsilon_2 \Lambda_1 H\right),
\end{aligned} \tag{5}
$$

$$
\begin{aligned}
\mathscr{P}_6 = &-b_2 + \lambda_1 \left(\frac{(1-\xi_2 u_4(t))\chi_1}{N} - (1-\xi_2 u_4(t))\Upsilon_1 - \psi \Upsilon_2 \right) S_U + \\
&\lambda_2 \left(\frac{(1-\xi_2 u_4(t))\chi_1}{N} - (1-\xi_2 u_4(t))\Upsilon_1 - (1-\xi_1 u_1(t))\Upsilon_2 \right) S_A \\
&+ \lambda_3 \left(\frac{(1-\xi_2 u_4(t))\chi_1}{N} - (1-\xi_2 u_4(t))\Upsilon_1 - \right. \\
&(1-\nu)(1-\xi_1 u_1(t))\Upsilon_2) V + \lambda_4 (\psi \Upsilon_2 S_U + \\
&(S_A + (1-\nu)V)(1-\xi_1 u_1(t))\Upsilon_2) + \left(\frac{(1-\xi_2 u_4(t))\chi_1}{N} \right. \\
&-(1-\xi_2 u_4(t))\Upsilon_1) C) + \lambda_5 \left(\frac{(1-\xi_2 u_4(t))\chi_1}{N} - \right. \\
&(1-\xi_2 u_4(t))\Upsilon_1) R - \lambda_6 \left(\frac{(1-\xi_2 u_4(t))\chi_1 (S_U + S_A + V + R)}{N} - \right. \qquad (6) \\
&(1-\xi_2 u_4(t))\Upsilon_1 (S_U + S_A + V + R) + (1-\xi_1 u_1(t))\Upsilon_2 \Lambda_1 H - \\
&\left. (\mu + \gamma_1) - \frac{(1-\xi_1 u_1(t))\chi_2 (C + \eta(C_H + C_A)) \Lambda_1}{N} \right) - \\
&\lambda_7 \left(\gamma_1 + \frac{(1-\xi_1 u_1(t))\chi_2 \Lambda_2}{N} - (1-\xi_1 u_1(t))\Upsilon_2 \Lambda_2 A \right) - \\
&\lambda_8 \left(\frac{(1-\xi_2 u_4(t))\chi_1 C}{N} - (1-\xi_2 u_4(t))\Upsilon_1 C - \right. \\
&\left. (1-\xi_1 u_1(t))\Upsilon_2 \Lambda_1 H + \frac{(1-\xi_1 u_1(t))\chi_2 (C + \eta(C_H + C_A)) \Lambda_1}{N} \right) + \\
&\lambda_9 (1-\xi_1 u_1(t))\Upsilon_2 \Lambda_2 A,
\end{aligned}
$$

$$
\begin{aligned}
\mathscr{P}_7 = &-b_3 + \lambda_1 \left(\frac{(1-\xi_2 u_4(t))\chi_1 \omega}{N} - (1-\xi_2 u_4(t))\Upsilon_1 - \psi \Upsilon_2 \right) S_U + \\
&\lambda_2 \left(\frac{(1-\xi_2 u_4(t))\chi_1 \omega}{N} - (1-\xi_2 u_4(t))\Upsilon_1 - (1-\xi_1 u_1(t))\Upsilon_2 \right) S_A + \\
&\lambda_3 \left(\frac{(1-\xi_2 u_4(t))\chi_1 \omega}{N} - (1-\xi_2 u_4(t))\Upsilon_1 - (1-\nu)(1-\xi_1 u_1(t))\Upsilon_2 \right) V + \\
&\lambda_4 \left(\psi \Upsilon_2 S_U + (S_A + (1-\nu)V)(1-\xi_1 u_1(t))\Upsilon_2 + \left(\frac{(1-\xi_2 u_4(t))\chi_1 \omega}{N} - \right. \right. \\
&(1-\xi_2 u_4(t))\Upsilon_1) C) + \lambda_5 \left(\frac{(1-\xi_2 u_4(t))\chi_1 \omega}{N} - (1-\xi_2 u_4(t))\Upsilon_1 \right) R - \qquad (7) \\
&\lambda_6 \left(\frac{(1-\xi_2 u_4(t))\chi_1 \omega (S_U + S_A + V + R)}{N} - \right. \\
&(1-\xi_2 u_4(t))\Upsilon_1 (S_U + S_A + V + R) + (1-\xi_1 u_1(t))\Upsilon_2 \Lambda_1 H) - \\
&\lambda_7 \left((1-\xi_1 u_1(t))\Upsilon_2 \Lambda_2 A - \frac{(1-\xi_1 u_1(t))\chi_2 (C + \eta(C_H + C_A)) \Lambda_2}{N} - \mu - \delta_2 \right) \\
&- \lambda_8 \left(\frac{(1-\xi_2 u_4(t))\chi_1 \omega C}{N} - (1-\xi_2 u_4(t))\Upsilon_1 C - (1-\xi_1 u_1(t))\Upsilon_2 \Lambda_1 H \right) - \\
&\lambda_9 \left(\frac{(1-\xi_1 u_1(t))\chi_2 (C + \eta(C_H + C_A)) \Lambda_2}{N} - (1-\xi_1 u_1(t))\Upsilon_2 \Lambda_2 A \right),
\end{aligned}
$$

$$
\begin{aligned}
\mathscr{P}_8 = & -b_4 + \lambda_1 \left(\frac{(1-\xi_2 u_4(t))\chi_1 \omega}{N} - (1-\xi_2 u_4(t))\Upsilon_1 + \frac{\psi \chi_1 \eta}{N} - \psi \Upsilon_2 \right) S_U + \\
& \lambda_2 \left(\frac{(1-\xi_2 u_4(t))\chi_1 \omega}{N} + \frac{(1-\xi_1 u_1(t))\chi_2 \eta}{N} - (1-\xi_2 u_4(t))\Upsilon_1 - \right. \\
& \left. (1-\xi_1 u_1(t))\Upsilon_2 \right) S_A + \lambda_3 \left(\frac{(1-\xi_2 u_4(t))\chi_1 \omega}{N} - (1-\xi_2 u_4(t))\Upsilon_1 + \right. \\
& \left. \frac{(1-\nu)(1-\xi_1 u_1(t))\chi_2 \eta}{N} - (1-\nu)(1-\xi_1 u_1(t))\Upsilon_2 \right) V + \\
& \lambda_4 \left(\frac{\psi \chi_2 \left(C + \eta(C_H + C_A)\right) S_U}{N^2} - \frac{\psi \chi_2 \eta S_U}{N} + \right. \\
& (S_A + (1-\nu)V)(1-\xi_1 u_1(t))\Upsilon_2 - \frac{(S_A + (1-\nu)V)(1-\xi_1 u_1(t))\chi_2 \eta}{N} + \\
& \left. \left(\frac{(1-\xi_2 u_4(t))\chi_1 \omega}{N} - (1-\xi_2 u_4(t))\Upsilon_1 \right) C \right) + \lambda_5 \left(\frac{(1-\xi_2 u_4(t))\chi_1 \omega}{N} - \right. \\
& \left. (1-\xi_2 u_4(t))\Upsilon_1 \right) R - \lambda_6 \left(\frac{(1-\xi_2 u_4(t))\chi_1 \omega (S_U + S_A + V + R)}{N} + \phi_2 - \right. \\
& (1-\xi_2 u_4(t))\Upsilon_1 (S_U + S_A + V + R) - \left(\frac{(1-\xi_1 u_1(t))\chi_2 \eta \Lambda_1}{N} - \right. \\
& \left. \left. (1-\xi_1 u_1(t))\Upsilon_2 \Lambda_1 \right) H \right) - \lambda_7 \left((1-\xi_1 u_1(t))\Upsilon_2 \Lambda_2 - \right. \\
& \left. \frac{(1-\xi_1 u_1(t))\chi_2 \eta \Lambda_2}{N} \right) A - \lambda_8 \left(\frac{(1-\xi_2 u_4(t))\chi_1 \omega C}{N} - (1-\xi_2 u_4(t))\Upsilon_1 C - \right. \\
& \left. (1-\xi_1 u_1(t))\Upsilon_2 \Lambda_1 H + \frac{(1-\xi_1 u_1(t))\chi_2 \eta \Lambda_1 H}{N} - (\phi_2 + \gamma_2 + \mu + \delta_1) \right) - \\
& \lambda_9 \left(\gamma_2 + \frac{(1-\xi_1 u_1(t))\chi_2 \eta \Lambda_2 A}{N} - (1-\xi_1 u_1(t))\Upsilon_2 \Lambda_2 A \right),
\end{aligned}
\tag{8}
$$

$$
\begin{aligned}
\mathscr{P}_9 = & -b_5 + \lambda_1 \left(\frac{(1-\xi_2 u_4(t))\chi_1\omega}{N} - (1-\xi_2 u_4(t))\Upsilon_1 + \frac{\psi\chi_1\eta}{N} - \psi\Upsilon_2 \right) S_U + \\
& \lambda_2 \left(\frac{(1-\xi_2 u_4(t))\chi_1\omega}{N} + \frac{(1-\xi_1 u_1(t))\chi_2\eta}{N} - (1-\xi_2 u_4(t))\Upsilon_1 - \right. \\
& \left. (1-\xi_1 u_1(t))\Upsilon_2 \right) S_A + \lambda_3 \left(\frac{(1-\xi_2 u_4(t))\chi_1\omega}{N} - (1-\xi_2 u_4(t))\Upsilon_1 + \right. \\
& \left. \frac{(1-\nu)(1-\xi_1 u_1(t))\chi_2\eta}{N} - (1-\nu)(1-\xi_1 u_1(t))\Upsilon_2 \right) V + \\
& \lambda_4 \left(\psi\Upsilon_2 S_U - \frac{\psi\chi_2\eta S_U}{N} + (S_A + (1-\nu)V)(1-\xi_1 u_1(t))\Upsilon_2 - \right. \\
& \frac{(S_A + (1-\nu)V)(1-\xi_1 u_1(t))\chi_2\eta}{N} + \\
& \left. \left(\frac{(1-\xi_2 u_4(t))\chi_1\omega}{N} - (1-\xi_2 u_4(t))\Upsilon_1 \right) C \right) + \lambda_5 \left(\frac{(1-\xi_2 u_4(t))\chi_1\omega}{N} - \right. \\
& \left. (1-\xi_2 u_4(t))\Upsilon_1 \right) R - \lambda_6 \left(\frac{(1-\xi_2 u_4(t))\chi_1\omega(S_U + S_A + V + R)}{N} - \right. \\
& (1-\xi_2 u_4(t))\Upsilon_1(S_U + S_A + V + R) - \left(\frac{(1-\xi_1 u_1(t))\chi_2\eta\Lambda_1}{N} - \right. \\
& \left. \left. (1-\xi_1 u_1(t))\Upsilon_2\Lambda_1 \right) H \right) - \lambda_7 \left(\phi_3 - \left((1-\xi_1 u_1(t))\Upsilon_2\Lambda_2 - \right. \right. \\
& \left. \left. \frac{(1-\xi_1 u_1(t))\chi_2\eta\Lambda_2}{N} \right) A \right) - \lambda_8 \left(\frac{(1-\xi_2 u_4(t))\chi_1\omega C}{N} - (1-\xi_2 u_4(t))\Upsilon_1 C \right. \\
& \left. -(1-\xi_1 u_1(t))\Upsilon_2\Lambda_1 H + \frac{(1-\xi_1 u_1(t))\chi_2\eta\Lambda_1 H}{N} \right) - \\
& \lambda_9 \left(\frac{(1-\xi_1 u_1(t))\chi_2\eta\Lambda_2 A}{N} - (1-\xi_1 u_1(t))\Upsilon_2\Lambda_2 A - (\phi_3 + \mu + \delta_3) \right).
\end{aligned} \tag{9}
$$

Where

$$
\Upsilon_1 = \frac{\xi_1 \left(H + \omega(A + C_H + C_A) \right)}{N^2}, and \quad \Upsilon_2 = \frac{\xi_2 \left(C + \eta(C_H + C_A) \right)}{N^2}
$$
